# Heritability and genome‐wide association study of blood pressure in Chinese adult twins

**DOI:** 10.1002/mgg3.1828

**Published:** 2021-09-29

**Authors:** Jiahao Chen, Weijing Wang, Zhaoying Li, Chunsheng Xu, Xiaocao Tian, Dongfeng Zhang

**Affiliations:** ^1^ Department of Epidemiology and Health Statistics Public Health College Qingdao University Qingdao China; ^2^ State Key Laboratory of Pathogen and Biosecurity Beijing Institute of Microbiology and Epidemiology Beijing P. R. China; ^3^ Qingdao Municipal Center for Disease Control and Prevention Qingdao Institute of Preventive Medicine Qingdao Shandong China

**Keywords:** blood pressure, GWAS, heritability, twins

## Abstract

**Background:**

Blood pressure (BP) is an independent and important factor for chronic diseases such as cardiovascular diseases and diabetes.

**Methods:**

We firstly conducted twin modeling analyses to explore the heritability of BP, including systolic blood pressure (SBP), diastolic blood pressure (DBP), pulse pressure (PP) and mean arterial pressure (MAP), and then performed genome‐wide association studies to explore the associated genomic loci, genes, and pathways.

**Results:**

A total of 380 Chinese twin pairs were included. The AE model containing additive genetic parameter (A) and unique/non‐shared environmental parameter (E) was the best fit model, with A accounting for 53.7%, 50.1%, 48.1%, and 53.3% for SBP, DBP, PP and MAP, respectively. No SNP was found to reach the genome‐wide significance level (*p* < 5 × 10^−8^), however, three, four, 14 and nine SNPs were found to exceed suggestive significance level (*p* < 1 × 10^−5^) for SBP, DBP, PP, and MAP, respectively. And after imputation, 46, 37, 91 and 61 SNPs were found to exceed the suggestive significance level for SBP, DBP, PP, and MAP, respectively. In gene‐based analysis, 53 common genes were found among SBP, DBP, PP, and MAP. In pathway enrichment analysis, 672, 706, 701, and 596 biological pathways were associated with SBP, DBP, PP, and MAP, respectively (*p* < 0.05).

**Conclusion:**

Our study suggests that BP is moderately heritable in the Chinese population and could be mediated by a series of genomic loci, genes, and pathways. Future larger‐scale studies are needed to confirm our findings.

## Graphical abstract

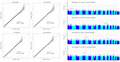

## 1 INTRODUCTION

Blood pressure (BP), as an important physiological index, is an independent and important factor for cardiovascular diseases (CVD) (Yang et al., 2012) which is one of the leading causes of mortality worldwide (Nitsa et al., 2018). In 2015, World Health Organization (WHO) reported that CVD could lead to more than 17.7 million deaths, accounting for 31% of global deaths (Roth et al., 2017). BP is a complex trait which can be affected by genetic and environmental factors (Wang et al., 2011). While comparing with the large number of studies on environmental factors for BP, the number of studies on genetic factors is relatively limited. Hence, it is necessary to explore the potential genetic factors. And it will be helpful for providing new clues for BP physiology and advancing our understanding of BP regulation.

At present, the magnitude of genetic sources of variance affecting BP level has been previously explored by several population studies. The results of heritability of BP were inconsistent, ranging from 25% to 60% (Bochud et al., 2005; Ehret, 2010; Gu et al., 2007; Kupper et al., 2005; Levy et al., 2000; Mitchell et al., 2005; Pilia et al., 2006; van Rijn et al., 2007; Rotimi et al., 1999). For different BP indexes, the heritability of systolic blood pressure (SBP), diastolic blood pressure (DBP), pulse pressure (PP), and mean arterial pressure (MAP) was 30%–45% (Levy et al., 2000; Pilia et al., 2006; van Rijn et al., 2007; Rotimi et al., 1999), 30%–60% (Gu et al., 2007; Levy et al., 2000; Pilia et al., 2006; van Rijn et al., 2007; Rotimi et al., 1999), 25%–55% (Bochud et al., 2005; Mitchell et al., 2005; van Rijn et al., 2007) and about 30% (Gu et al., 2007; Mitchell et al., 2005), respectively. Additionally, some genome‐wide association studies (GWASs) reported that several SNPs such as rs2681472, rs11067763 (Levy et al., 2009; Lu et al., 2015) might be associated with SBP, rs2681472, rs2681492 and rs17030613 (Hong et al., 2010; Kato et al., 2011) with DBP, rs11466481, rs117386367, and rs13002573 (Sofer et al., 2017; Wain et al., 2011) with PP, rs1446468, rs319690, and rs9366626 (Sofer et al., 2017; Wain et al., 2011) with MAP. And their corresponding genes were also associated with SBP, DBP, PP, and MAP, respectively (Hong et al., 2010; Kato et al., 2011; Levy et al., 2009; Lu et al., 2015; Sofer et al., 2017; Wain et al., 2011).

Although certain BP‐associated genetic loci and genes have been found, they could only explain a part of the genetic influence. And life style, hereditary characteristics and allele frequencies of the Chinese population are different from other ethnic populations worldwide. Hence, there still are some potential genetic loci and genes remained to be explored.

Due to the genetic relatedness, twin pairs studies could control the genetic effects on disease risk, thus they have a higher power in the genetic study, especially for human complex diseases (Tan et al., 2017). Therefore, in this GWAS based on a sample of 380 Chinese twin pairs, we aimed to explore the genetic effect on BP (SBP, DBP, PP and MAP) and investigate the promising genetic loci, genes, and pathways.

## 2 MATERIALS AND METHODS

### 2.1 Ethics statement

Helsinki Declaration was followed and this study was approved by the Regional Ethics Committee of the Institutional Review Committee of Qingdao CDC. Written informed consents were signed by everyone.

### 2.2 Twin samples collection

The process of collecting twin sample was conducted by Qingdao Twin Registry, and research recruitment details could be found in previous studies (Xu, Zhang, Tian, Duan, et al., 2017; Xu, Zhang, Tian, Wu, et al., 2017). The following exclusion criteria were applied: (1) participants who were pregnant or breastfeeding; (2) participants who took medications affecting blood pressure level; (3) the data of co‐twin pairs were incomplete. Finally, 380 twin pairs were included in this study, and 243 monozygotic (MZ) twin pairs and 137 dizygotic (DZ) twin pairs were used to conduct heritability analysis and the 137 DZ twin pairs were further used in GWAS. The zygosity was determined by gender, ABO blood type, and 16 multiple short tandem sequence repeat DNA markers (Becker et al., 1997; Tomsey et al., 2001).

### 2.3 Phenotypes

Participants firstly rested quietly in a sitting position for five minutes, and then their blood pressure was measured three times using a mercurial table stand model sphygmomanometer. SBP and DBP were obtained from sphygmomanometer. PP and MAP were calculated by SBP and DBP (PP = SBP − DBP; MAP = 1/3 SBP + 2/3 DBP).

### 2.4 Genotyping, quality control and imputation

All 137 DZ pairs were genotyped using the Illumina's InfiniumOmni2.5Exome‐8v1.2 BeadChip platform (Illumina). The following strict genotype quality control procedures were adopted: (1) call rate >0.98; (2) locus missing <0.05; (3) minor allele frequency (MAF) >0.05; (4) and Hardy‐Weinberg Equilibrium (HWE) >1 × 10^−4^. In the end, 1,364,336 SNPs were included in the subsequent GWAS analysis.

IMPUTE2 software (Marchini et al., 2007) was used to impute un‐typed SNPs following the linkage disequilibrium (LD) information from the East Asia 1000 Genomes Project Phase 3 reference panel (Auton et al., 2015). And the following criteria were used to screen the imputed data: (1) MAF >0.05; (2) HWE >1 × 10^−4^; (3) and *R* ^2^ > 0.6. In the end, 7,405,822 SNPs were included to further analysis.

### 2.5 Statistical analysis

#### 2.5.1 Heritability

SPSS 22.0 was used to prepare and describe data, and Mx program was used to perform genetic analysis. Pearson's product‐moment correlation coefficients were calculated to evaluate twin pair phenotypic correlations. If the correlation coefficient of MZ twins was statistically higher than that of DZ twins, indicating significant genetic effects exiting in BP variance.

The source of phenotypic variance was made up of several different parts: additive genetic effect (A), dominant genetic effect (D), common or shared environmental effect (C) and unique/non‐shared environmental variance (E). The fitting model was determined by comparing the correlation coefficients of MZ and DZ. If r_MZ_ was greater than two times of r_DZ_, the ADE model was adopted; otherwise, the ACE model was adopted. Then, the optimal model was determined by the results of likelihood ratio chi‐square (*p* > 0.05) and Akaike's Information Criterion (AIC) value. Age, gender, and body mass index (BMI) were adjusted in all models. And Mx software was also used to calculate the power of twin pairs for additive genetic influences (>90%).

#### 2.5.2 GWAS

##### 2.5.2.1 SNPs‐based analysis

Genome‐wide efficient mixed‐model association (GEMMA; Zhou & Stephens, 2012) was used to test the association between BP and SNP genotypes, with age, gender and BMI being adjusted. It fits a Bayesian sparse linear mixed model using Markov chain Monte Carlo for estimating the proportion of variance in phenotypes explained by typed genotypes, predicting phenotypes, and identifying associated markers by jointly modeling all markers while controlling for population structure. *p* < 5 × 10^−8^ was defined as conventional genome‐wide significance level, and *p* < 1 × 10^−5^ was defined as suggestive level (Dudbridge & Gusnanto, 2008). Quantile‐quantile (Q‐Q) plot was used to assess whether there was stratification effect in the population. Manhattan plot was used to represent the value (−log_10_ *p*) of each SNP on each chromosome. The base pair position is based on the Genome Reference Consortium Human Build 38 (GRCh38).

##### 2.5.2.2 Gene‐based analysis

Versatile Gene‐based Association Study‐2 (VEGAS2) was used to perform gene‐based analysis. In VEGAS2, all SNPs were integrated into one gene to increase the intensity of correlation. One thousand genomes data were used to simulate the correlation between blood pressure and SNPs on autosomal and chromosome X (J. Z. Liu et al., 2010; Mishra & Macgregor, 2015). SNPs data from the “1000G East ASIAN Population” was used as reference. Because 19,001 genes were evaluated, so statistical significance was adjusted to *p* < 2.63 × 10^−6^ (0.05/19,001).

##### 2.5.2.3 Pathway enrichment analysis

PASCAL was used to calculate pathway‐scored (Julia et al., 2018; Lamparter et al., 2016). In PASCAL, genetic markers SNPs were firstly mapped to genes, and all gene scores in the pathway were calculated. Then, all gene scores in the pathway were integrated as the pathway scores. Empirical values and chi‐square values were used to evaluate high‐score gene pathways in this study. All pathways and related gene annotations were obtained from Reactome, KEGG and BioCarta.

## 3 RESULTS

### 3.1 Heritability

The mean value (M) ± standard deviation (SD) of all participants age was 51.52 ± 7.62 years, and that of 137 DZ twin pairs was 50.99 ± 7.18 years. The M ± SD of SBP, DBP, PP, and MAP level for all subjects was 130.76 ± 20.09 mmHg, 83.06 ± 10.88 mmHg, 47.70 ± 14.66 mmHg, and 98.96 ± 12.87 mmHg, respectively (Table 1). After adjusting for age, gender and BMI, the correlations of MZ twin pairs for SBP (*r*_MZ_ = 0.53, 95% CI: 0.44–0.61), DBP (*r*_MZ_ = 0.50, 95% CI: 0.40–0.61), PP (*r*_MZ_ = 0.47, 95% CI: 0.37–0.56), and MAP (*r*_MZ_ = 0.53, 95% CI: 0.44–0.61) outweighed that of DZ twin pairs for SBP (*r*_MZ_ = 0.30, 95% CI: 0.14–0.44), DBP (*r*_MZ_ = 0.28, 95% CI: 0.11–0.43), PP (*r*_MZ_ = 0.29, 95% CI: 0.14–0.42), and MAP (*r*_MZ_ = 0.27, 95% CI: 0.09–0.41), indicating the genetic effects on SBP, DBP, PP, and MAP (Table 2). And the correlations of MZ twin for SBP, DBP, PP, and MAP were less than twice that of DZ twin, suggesting ACE model was the appropriate fitting model.

**TABLE 1:** Descriptive statistics for subjects in monozygotic and dizygotic twin pairs

		N	Age (years)	BMI (Kg/m^2^)	SBP (mm Hg)	DBP (mm Hg)	PP (mm Hg)	MAP (mm Hg)
MZ	Male	230	52.95 ± 9.05	24.16 ± 3.11	135.84 ± 19.03	86.13 ± 11.24	49.71 ± 14.90	102.70 ± 12.47
	Female	256	50.81 ± 6.47	24.21 ± 3.52	124.42 ± 19.25	79.21 ± 9.84	45.21 ± 13.27	94.28 ± 12.20
	Total	486	51.82 ± 7.86	24.19 ± 3.33	129.80 ± 19.96	82.47 ± 11.07	47.33 ± 14.23	98.25 ± 13.02
DZ	Male	140	50.84 ± 7.32	24.65 ± 3.02	135.25 ± 19.38	86.15 ± 10.65	49.10 ± 14.81	102.52 ± 12.34
	Female	134	51.15 ± 7.05	24.50 ± 3.23	129.57 ± 20.74	81.99 ± 9.88	47.59 ± 15.97	97.85 ± 12.32
	Total	274	50.99 ± 7.18	24.58 ± 3.12	132.45 ± 20.23	84.10 ± 10.47	48.36 ± 15.38	100.22 ± 12.53
Total	Male	370	52.15 ± 8.49	24.35 ± 3.08	135.61 ± 19.14	86.14 ± 11.01	49.48 ± 14.85	102.63 ± 12.40
	Female	390	50.93 ± 6.67	24.31 ± 3.42	126.21 ± 19.91	80.17 ± 9.93	46.03 ± 14.29	95.52 ± 12.34
	Total	760	51.52 ± 7.62	24.33 ± 3.26	130.76 ± 20.09	83.06 ± 10.88	47.70 ± 14.66	98.96 ± 12.87

Note

All data are expressed in mean ± standard deviation.

Abbreviations: BMI, body mass index; DBP, diastolic blood pressure; DZ, dizygotic; MAP, mean arterial pressure; MZ, monozygotic; PP, pulse pressure; SBP, systolic blood pressure.

**TABLE 2:** Phenotypic correlation coefficients (95% confidence intervals) with covariates’ effects in MZ and DZ twin pairs

	Model	Correlation		Model testing			
		MZ	DZ	−2LL	*df*	*χ* ^2^	*p*
SBP	Base	0.53 (0.44–0.61)	0.30 (0.14–0.44)	1893.3	753	—	—
	Drop age	0.57 (0.48–0.64)	0.36 (0.20–0.49)	1936.6	754	43.3	<0.01
	Drop sex	0.57 (0.48–0.64)	0.36 (0.20–0.49)	1922.6	754	29.3	<0.01
	Drop BMI	0.53 (0.44–0.61)	0.31 (0.14–0.44)	1946.5	754	53.2	<0.01
DBP	Base	0.50 (0.40–0.58)	0.28 (0.11–0.43)	1943.9	753	—	—
	Drop age	0.50 (0.40–0.58)	0.28 (0.11–0.43)	1944.7	754	0.8	0.37
	Drop sex	0.54 (0.45–0.62)	0.34 (0.17–0.48)	1984.3	754	40.4	<0.01
	Drop BMI	0.48 (0.38–0.57)	0.30 (0.14–0.44)	2003.2	754	59.4	<0.01
PP	Base	0.47 (0.37–0.56)	0.29 (0.14–0.42)	1956.7	753	—	—
	Drop age	0.53 (0.44–0.62)	0.37 (0.23–0.49)	2022.5	754	65.7	<0.01
	Drop sex	0.48 (0.38–0.57)	0.30 (0.15–0.43)	1961.9	754	5.1	0.023
	Drop BMI	0.48 (0.38–0.57)	0.29 (0.14–0.42)	1971.2	754	14.5	<0.01
MAP	Base	0.53 (0.44–0.61)	0.27 (0.09–0.41)	1912.3	753	—	—
	Drop age	0.55 (0.45–0.62)	0.29 (0.12–0.44)	1928.6	754	16.3	<0.01
	Drop sex	0.57 (0.49–0.65)	0.34 (0.17–0.48)	1951.6	754	39.2	<0.01
	Drop BMI	0.52 (0.43–0.60)	0.28 (0.11–0.42)	1979.6	754	67.2	<0.01

Abbreviations: −2LL, −2 Log Likelihood; BMI, body mass index; DBP, diastolic blood pressure; *df*, degree of freedom; DZ, dizygotic; MAP, mean arterial pressure; MZ, monozygotic; PP, pulse pressure; SBP, systolic blood pressure.

Then results of likelihood ratio chi‐square and AIC were applied to choose the best nested model. Finally, AE model was the best fitting model for SBP, DBP, PP and MAP. In AE model for SBP, A accounted for 53.70% (95% CI: 44.90%–61.40%) and E for 46.30% (95% CI: 38.60%–55.10%). In AE model for DBP, A accounted for 50.10% (95% CI: 40.80%–58.20%) and E for 50.00% (95% CI: 41.90%–59.20%). In AE model for PP, A accounted for 48.10% (95% CI: 38.40%–56.60%) and E for 51.90% (95% CI: 43.40%–61.60%). In AE model for MAP, A accounted for 53.30% (95% CI: 44.30%–61.10%) and E for 46.70% (95% CI: 38.90%–55.70%) (Table 3).

**TABLE 3:** Model fit and proportion of variance for SBP, DBP, PP, MAP level accounted by genetic and environmental parameters

	Model	A	C	E	−2LL	*df*	AIC	χ*^2^*	*p*
SBP	ACE	46.90 (14.60–61.30)	6.50 (0.00–35.20)	46.60 (38.70–55.90)	1893.3	753	387.3		
	**AE**	**53.70 (44.90–61.40)**	**—**	**46.30 (38.60–55.10)**	**1893.5**	**754**	**385.5**	**0.2**	**0.69**
	CE	**—**	45.30 (36.90–53.10)	54.70 (46.90–63.10)	1901.8	754	393.8	8.5	<0.01
DBP	ACE	43.30 (9.30–58.00)	6.40 (0.00–36.70)	50.30 (42.00–59.90)	1943.9	753	437.9		
	**AE**	**50.10 (40.80–58.20)**	**—**	**50.00 (41.90–59.20)**	**1944.0**	**754**	**436.0**	**0.1**	**0.71**
	CE	**—**	42.70 (34.00–50.70)	57.30 (49.30–66.00)	1950.3	754	442.3	6.4	0.011
PP	ACE	36.60 (4.00–56.10)	10.60 (0.00–38.30)	52.80 (43.80–63.20)	1956.7	753	450.7		
	**AE**	**48.10 (38.40–56.60)**	**—**	**51.90 (43.40–61.60)**	**1957.2**	**754**	**449.2**	**0.5**	**0.49**
	CE	**—**	39.90 (31.10–48.10)	60.10 (52.00–68.90)	1961.6	754	453.6	4.9	0.027
MAP	ACE	53.30 (19.90–61.10)	0.00 (0.00–30.10)	46.70 (38.90–55.90)	1912.3	753	406.3		
	**AE**	**53.30 (44.30–61.10)**	**—**	**46.70 (38.90–55.70)**	**1912.3**	**754**	**404.3**	**0.0**	**1.00**
	CE	**—**	44.50 (35.90–52.30)	55.50 (47.70–64.10)	1922.7	754	414.7	10.3	<0.01

Abbreviations: −2LL, −2 Log Likelihood; A, additive genetic influence; AIC, Akaike's information criterion; C, common or shared environmental influence; DBP, diastolic blood pressure; *df*, degree of freedom; E, unique or non‐shared environmental influence; MAP, mean arterial pressure; PP, pulse pressure; SBP, systolic blood pressure.

Bold indicates that the model is the best fit model.

### 3.2 GWAS

#### 3.2.1 SNPs‐based analysis

**FIGURE 1:**
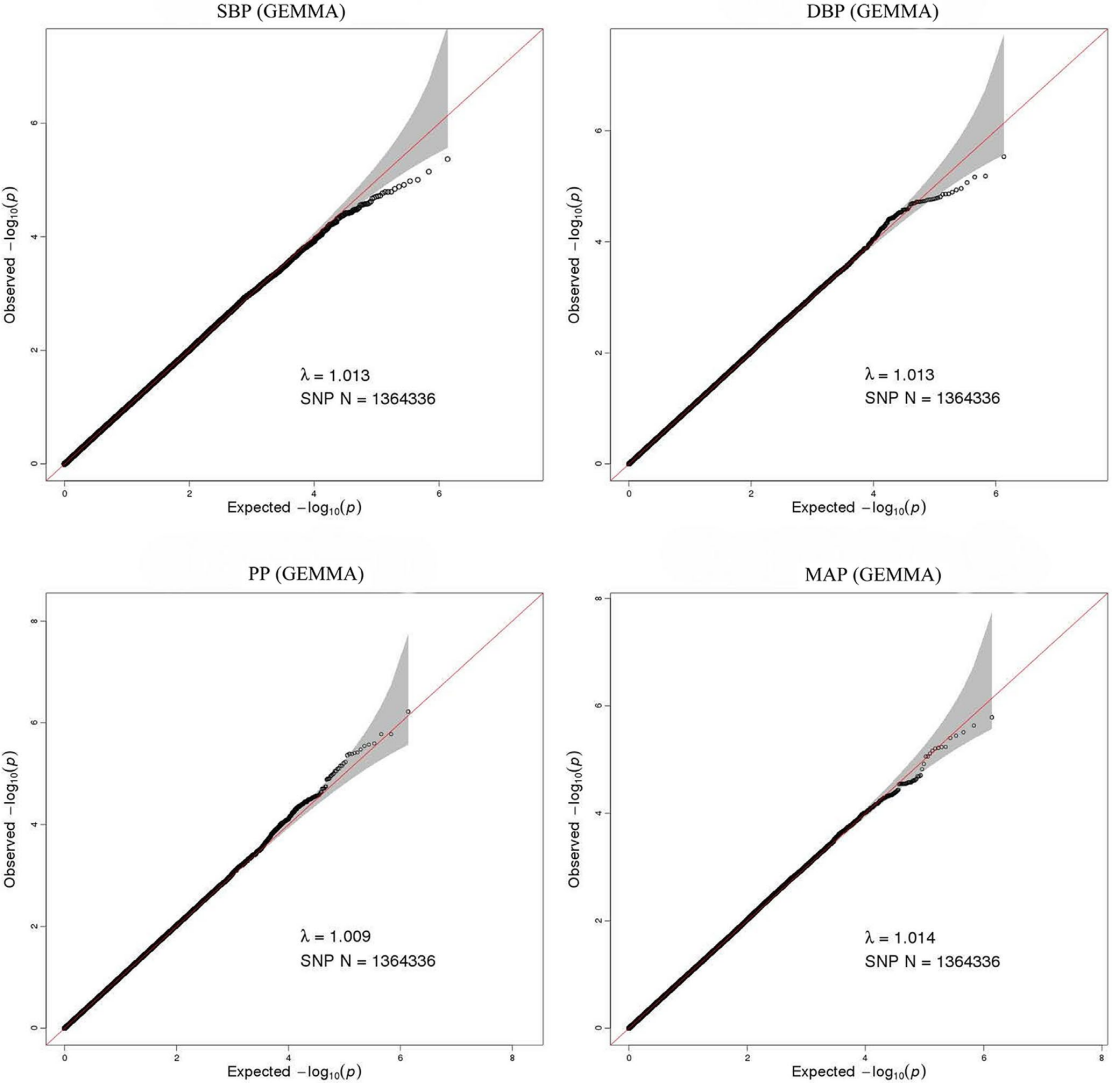
Quantile‐quantile plot for quality control check and visualizing crude association for genome‐wide association study of SBP, DBP, PP, and MAP. The x‐axis shows the −log^10^ of expected *p*‐values of association from chi‐square distribution and the y‐axis shows the −log^10^ of *p*‐values from the observed chi‐square distribution. The black dots represent the observed data, and the red line is the expectation under the null hypothesis of no association

**FIGURE 2:**
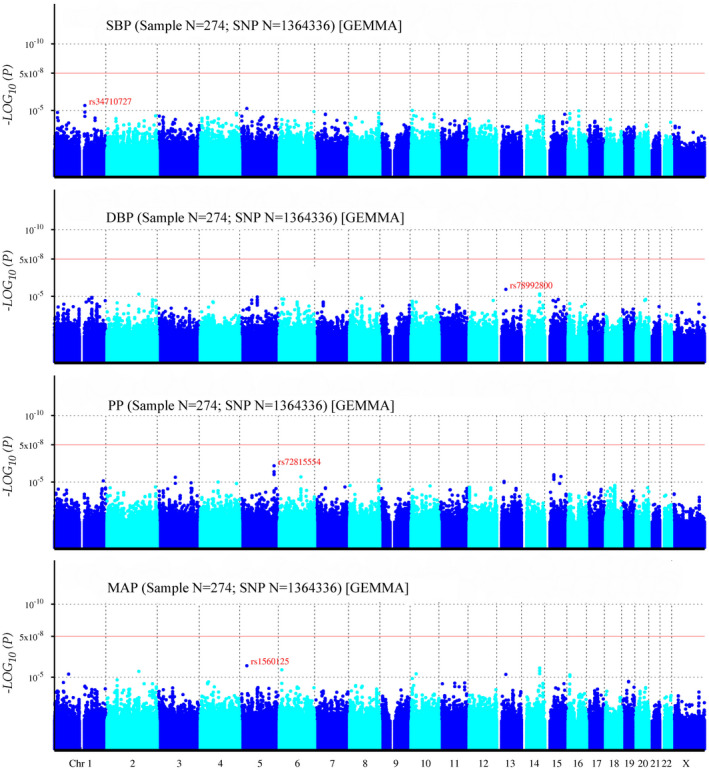
Manhattan plot for genome‐wide association study of SBP, DBP, PP, and MAP. The x‐axis shows the numbers of autosomes and the X chromosome, and the y‐axis shows the *p*‐values for statistical significance. The dots represent the SNPs. None of the SNPs reached the genome‐wide significance level (*p* < 5 × 10^−8^)

In 137 DZ twin pairs, a total of 1,364,336 SNPs was included into GWAS of BP. The Q‐Q plots of SBP, DBP, PP, and MAP illustrated the correction between observed and expected GWAS *p*‐values (Figure 1). The value of genomic inflation factor (*λ*) for SBP, DBP, PP and MAP was 1.013, 1.013, 1.009, and 1.014, respectively, indicating that there were no population stratification effects. And the slight deviation in the upper right tail in the four Q‐Q plots indicated the existences of weak associations. Even no SNPs reached the genome‐wide significance level as the Manhattan plots (Figure 2) shown, some SNPs exceeded the threshold of the suggestive significance level.

For SBP, three SNPs surpassed the threshold of the suggestive significance level (Table 4), and the strongest associated SNP was rs34710727 (*p* = 4.28 × 10^−6^), locating at chromosome 1 and positioning closed to long intergenic non‐protein coding RNA 624 gene (*LINC00624*, OMIM accession number: NA).

**TABLE 4:** Summary of SNPs with *p*‐value <1 × 10^−5^ for association with SBP, DBP, PP, and MAP in genome‐wide association study

SNP	Level	CHR	BP	*p*‐value	Closest genes or genes	Official full name
rs34710727	SBP	1	146,997,592	4.28E‐06	LINC00624	Long intergenic non‐protein coding RNA 624
rs1560125	SBP	5	29,418,130	7.12E‐06	LINC02064	Long intergenic non‐protein coding RNA 2064
rs11256258	SBP	10	6,033,415	9.93E‐06	IL15RA	Interleukin 15 receptor subunit alpha
rs78992800	DBP	13	38,939,829	2.94E‐06	UFM1	Ubiquitin fold modifier 1
rs57037058	DBP	14	82,437,952	6.59E‐06	EIF3LP1	Eukaryotic translation initiation factor 3 subunit L pseudogene 1
rs34326233	DBP	2	153,770,846	6.83E‐06	UBQLN4P2	Ubiquilin 4 pseudogene 2
rs72695476	DBP	14	82,429,884	8.60E‐06	EIF3LP1	Eukaryotic translation initiation factor 3 subunit L pseudogene 1
rs72815554	PP	5	160,995,760	6.03E‐07	GABRB2	Gamma‐aminobutyric acid type A receptor beta2 subunit
rs6881515	PP	5	160,911,968	1.66E‐06	GABRB2	Gamma‐aminobutyric acid type A receptor beta2 subunit
rs12153198	PP	5	160,915,124	1.66E‐06	GABRB2	Gamma‐aminobutyric acid type A receptor beta2 subunit
rs72815551	PP	5	160,995,192	2.54E‐06	GABRB2	Gamma‐aminobutyric acid type A receptor beta2 subunit
rs11956795	PP	5	160,983,125	2.68E‐06	GABRB2	Gamma‐aminobutyric acid type A receptor beta2 subunit
rs75457329	PP	6	104,716,728	4.09E‐06	LOC105377917	Uncharacterized
rs9875783	PP	3	82,477,805	4.35E‐06	LINC02008	Long intergenic non‐protein coding RNA 2008
rs67701708	PP	8	140,916,796	7.01E‐06	TRAPPC9	Trafficking protein particle complex 9
rs1075493	PP	8	140,917,457	7.01E‐06	TRAPPC9	Trafficking protein particle complex 9
rs13266333	PP	8	140,913,144	7.90E‐06	TRAPPC9	Trafficking protein particle complex 9
rs35440803	PP	1	236,620,413	8.08E‐06	EDARADD	EDAR associated death domain
rs9550532	PP	13	30,723,602	8.91E‐06	LINC00384	Long intergenic non‐protein coding RNA 384
rs913905	PP	13	30,720,425	8.93E‐06	LINC00384	Long intergenic non‐protein coding RNA 384
rs4235077	PP	4	86,026,327	9.95E‐06	RN7SKP48	RN7SK pseudogene 48
rs1560125	MAP	5	29,418,130	1.64E‐06	LINC02064	Long intergenic non‐protein coding RNA 2064
rs72695476	MAP	14	82,429,884	2.32E‐06	EIF3LP1	Eukaryotic translation initiation factor 3 subunit L pseudogene 1
rs6927364	MAP	6	12,595,275	3.11E‐06	LINC02530	Long intergenic non‐protein coding RNA 2530
rs57037058	MAP	14	82,437,952	3.60E‐06	EIF3LP1	Eukaryotic translation initiation factor 3 subunit L pseudogene 1
rs34326233	MAP	2	153,770,846	3.97E‐06	UBQLN4P2	Ubiquilin 4 pseudogene 2
rs72695477	MAP	14	82,433,929	5.81E‐06	EIF3LP1	Eukaryotic translation initiation factor 3 subunit L pseudogene 1
rs1888656	MAP	10	24,833,705	5.86E‐06	KIAA1217	KIAA1217
rs7526959	MAP	1	68,566,576	6.06E‐06	WLS	Wnt ligand secretion mediator
					GNG12‐AS1	GNG12, DIRAS3 and WLS antisense RNA 1
rs78992800	MAP	13	38,939,829	6.25E‐06	UFM1	Ubiquitin fold modifier 1

Abbreviations: BP, base pair SNPs information was from Build 38 (GRCh38); CHR, chromosome; DBP, diastolic blood pressure; MAP, mean arterial pressure; PP, pulse pressure; SBP, systolic blood pressure.

Among four SNPs exceeding the threshold of the suggestive significance level of DBP (Table 4), rs78992800 was the strongest associated SNP with DBP (*p* = 2.94 × 10^−6^), positioning closed to ubiquitin fold modifier 1 gene (*UFM1*, chromosome 13, OMIM accession number: 610553), which was important to cardiac homeostasis and blood regulation. SNPs rs57037058 and rs72695476 were found near eukaryotic translation initiation factor 3 subunit L pseudogene 1 gene (*EIF3LP1*, chromosome 14, OMIM accession number: NA).

A total of 14 SNPs was found to go beyond the threshold of the suggestive significance level of PP (Table 4). Five SNPs (rs72815554, rs6881515, rs12153198, rs72815551, and rs11956795) were closed to the gamma‐aminobutyric acid type A receptor subunit beta2 gene (*GABRB2*, chromosome 5, OMIM accession number: 600232). Among them, rs72815554 was the strongest associated SNP (*p* = 6.03 × 10^−7^). And on chromosome 8, trafficking protein particle complex subunit 9 gene (*TRAPPC9*, OMIM accession number: 611969) was an important gene related to BP, and three SNPs rs67701708, rs1075493, and rs13266333 were found to near it.

Nine SNPs exceeded the threshold of suggestive significance level of MAP (Table 4). The strongest related SNP (rs1560125; *p* = 1.64 × 10^−6^) located at chromosome 5 and long intergenic non‐protein coding RNA 2064 gene (*LINC02064*, OMIM accession number: NA). And on chromosome 14, three SNPs rs72695476, rs57037058, and rs72695477 were found to near the *EIF3LP1* gene.

#### 3.2.2 Imputation

**FIGURE 3:**
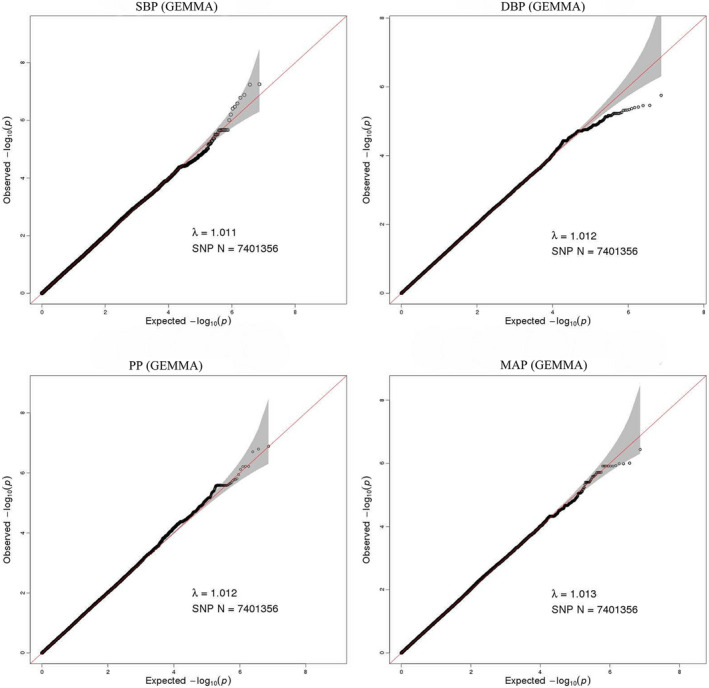
Quantile‐quantile plot for quality control check and visualizing crude association for genome‐wide association study of SBP, DBP, PP, and MAP. The x‐axis shows the −log^10^ of expected *p*‐values of association from chi‐square distribution and the y‐axis shows the −log^10^ of *p*‐values from the observed chi‐square distribution. The black dots represent the observed data, and the red line is the expectation under the null hypothesis of no association (after imputation)

**FIGURE 4:**
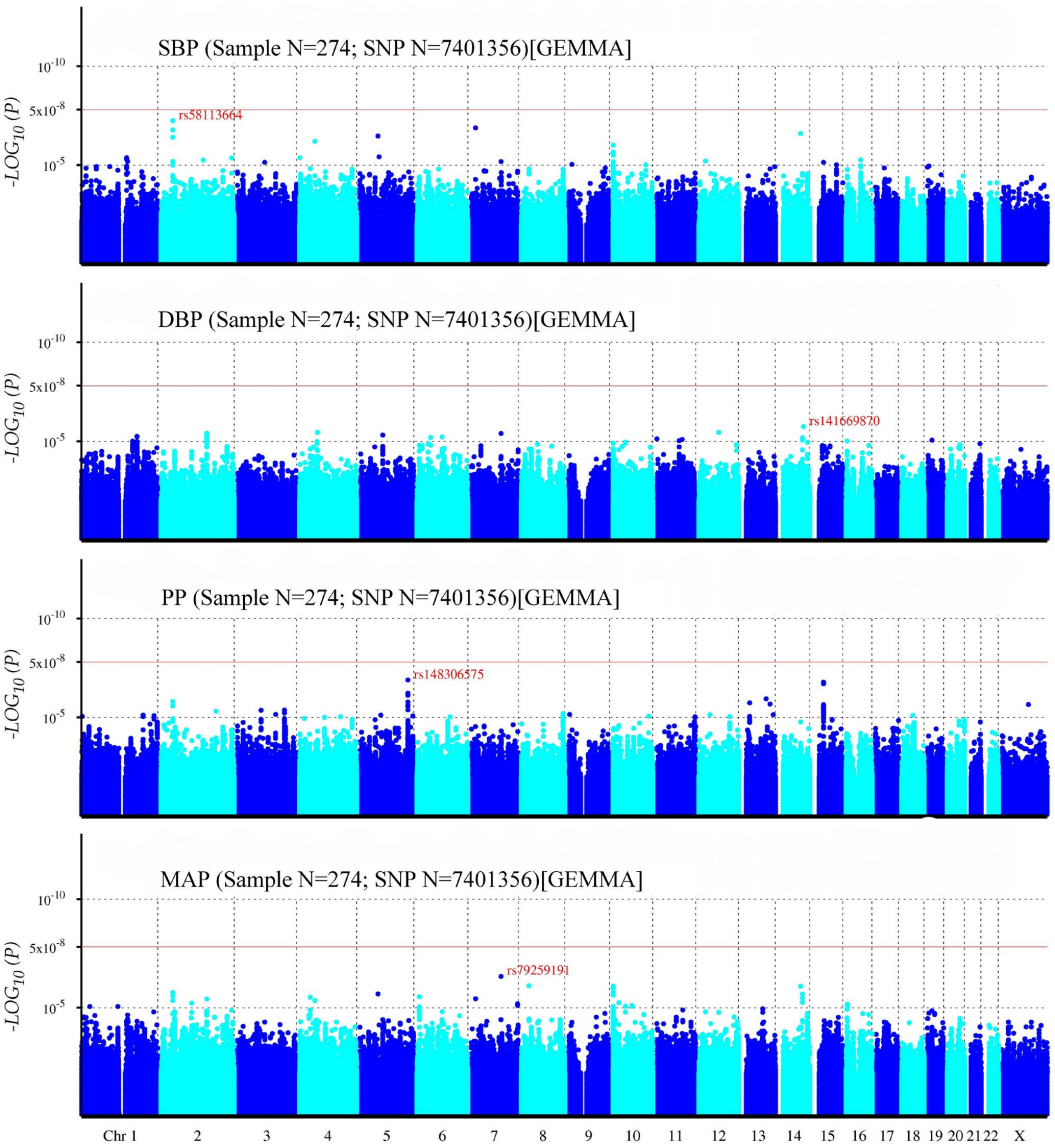
Manhattan plot for genome‐wide association study of SBP, DBP, PP, and MAP. The x‐axis shows the numbers of autosomes and the X chromosome, and the y‐axis shows the *p*‐values for statistical significance. The dots represent the SNPs. None of the SNPs reached the genome‐wide significance level (*p* < 5 × 10^−8^) (after imputation)

Typed SNPs were imputed to identify new risk variants and 1,000 Genomes Project Phase 3 was used as the reference panel. The post‐imputation Q–Q plots of SBP, DBP, PP and MAP illustrated there were no population stratification effects (Figure 3). No SNP was found to reach the genome‐wide significance level in post‐imputation Manhattan plots of SBP, DBP, PP, and MAP (Figure 4). While, 46, 37, 91, and 61 SNPs were found to exceed the threshold of suggestive significance level for SBP, DBP, PP, and MAP, respectively. The strongest associated SNPs were rs58113664, rs141669870, rs148306575, and rs79259191 for SBP, DBP, PP, and MAP, respectively (Tables [Link], [Link], [Link], [Link]).

#### 3.2.3 Gene‐based analysis

No gene was found to achieve genome‐wide significance level in gene‐based analysis. So, we explored the genes most closely related to blood pressure, and the top 20 genes of SBP, DBP, PP and MAP were shown in Tables [Link], [Link], [Link], [Link]. And 53 common genes were found among SBP, DBP, PP and MAP (*p* < 0.05), including thyroid hormone receptor beta (*THRB*, OMIM accession number: 190160), proteasome 20S subunit beta 3 (*PSMB3*, OMIM accession number: 602176), olfactory receptor family 8 subfamily D member 1 (*OR8D1*, OMIM accession number: NA) and so on (Table S9).

#### 3.2.4 Pathway enrichment analysis

In our study, 672, 706, 701, and 596 pathways were found to be associated with SBP, DBP, PP, and MAP, respectively (*p* < 0.05). The top 20 pathways of SBP, DBP, PP and MAP were shown in Tables [Link], [Link], [Link], [Link]. Among them, some pathways could be explained reasonably, such as dilated cardiomyopathy, hormone ligand binding receptors, GAB1 signalosome, platelet aggregation plug formation and so on. And 146 common pathways were found among SBP, DBP, PP, and MAP, including BIOCARTA_EGFR_SMRTE_PATHWAY, KEGG_DILATED_CARDIOMYOPATHY, REACTOME_GAB1_SIGNALOSOME, and so on.

## 4 DISCUSSION

### 4.1 Heritability

In our study containing 380 twin pairs, the correlation coefficient of SBP, DBP, PP and MAP in MZ twins was higher than that of DZ twins, reflecting the existences of significant genetic effect on BP. The AE model was the best fit model for SBP, DBP, PP and MAP, with A accounting for 53.7%, 50.10%, 48.10%, and 53.30%, respectively, which were consistent with previous studies (Ehret, 2010; Gu et al., 2007; Kupper et al., 2005; Levy et al., 2000; Pilia et al., 2006; van Rijn et al., 2007; Rotimi et al., 1999). At the same time, among East Asian populations, the research on blood pressure heritability has been mainly concentrated in China, and some studies have been conducted in South Korea (Jiang et al., 2012; Kim et al., 2015; Sung et al., 2009; Wu et al., 2011). In general, the heritability of blood pressure in East Asian populations was around 20%‐60%, which is also consistent with our findings, indicating our conclusion is credible and stable.

### 4.2 GWAS

#### 4.2.1 SNP‐based analysis

##### SBP

Though genome‐wide significant SNP was found in our study, we found three associated SNPs, rs34710727 located on chromosome 1, rs1560125 located on chromosome 5 and rs11256258 located on chromosome 10. They correspond to *LINC00624*, *LINC02064*, and interleukin 15 receptor subunit alpha gene (*IL15RA*, OMIM accession number: 601070), respectively. *IL15RA* gene corresponds to IL‐15Rα, which is an important subunit of IL‐15. At present, no research has found the exact relationship between IL‐15 or IL‐15Rα and BP. But some studies (Kivisakk et al., 1998; Liu et al., 2000; McInnes et al., 1996) have found the proinflammatory effect of IL‐15 in some diseases, such as multiple sclerosis, inflammatory bowel disease and rheumatoid arthritis. Inflammation plays an important role in regulating BP and hypertension, so IL‐15 or IL‐15Rα could also have effects on BP and hypertension. But further researches need to be conducted to prove this possible relationship.

##### DBP

No genome‐wide significant SNP was found in our study, but we found four associated SNPs, rs78992800 located on chromosome 13, rs57037058 located on chromosome 14, rs34326233 located on chromosome 2 and rs72695476 located on chromosome 14. They correspond to *UFM1*, *EIF3LP1*, ubiquilin 4 pseudogene 2 (*UBQLN4P2*, OMIM accession number: NA) and *EIF3LP1* gene, respectively. Study conducted by Li et al. (2018) found that *UFM1* was important to cardiac homeostasis by regulating endoplasmic reticulum function. Another study (Li, Zhang, et al., 2017) found that there was a relation between *UFM1* and endothelial cells. And it plays an important role in vascular remodeling (Su et al., 2018). These evidences suggested that *UFM1* might have an effect on blood pressure regulation.

##### PP

Fourteen SNPs were found to be related to PP. These 14 SNPs correspond to *GABRB2*, *LOC105377917*, long intergenic non‐protein coding RNA 2008 (*LINC02008*, OMIM accession number: NA), *TRAPPC9*, EDAR associated death domain (*EDARADD*, OMIM accession number: 606603), long intergenic non‐protein coding RNA 384 (*LINC00384*, OMIM accession number: NA) and RN7SK pseudogene 48 (*RN7SKP48*, OMIM accession number: NA). A study (Sung et al., 2015) about Framingham Heart Study founded that *TRAPPC9* was associated with blood pressure. And *TRAPPC9* was proved to be related to stroke in study conducted among Japanese (Yoshida et al., 2010). So *TRAPPC9* might play an important role in regulating pulse pressure by affecting cardiovascular system and blood pressure, but this possible relationship needs to be proved by further researches.

##### MAP

No genome‐wide significant SNP was found in our study, but we found nine associated SNPs. These nine SNPs correspond to *LINC02064*, *EIF3LP1*, long intergenic non‐protein coding RNA 2530 (*LINC02530*, OMIM accession number: NA), *UBQLN4P2*, *KIAA1217* (OMIM accession number: 617367), Wnt ligand secretion mediator (*WLS*, OMIM accession number: 611514), GNG12, DIRAS3 and WLS antisense RNA 1 (*GNG12*‐*AS1*, OMIM accession number: 611406) and *UFM1* gene. The effect of *UFM1* on cardiovascular system have been discussed in DBP. Except for *UFM1*, other genes have not been found to be related to cardiovascular system, more studies might need to be conducted to find out their relationship.

We further compared significant SNP (*p* < 0.05) in our results with that of previous genome‐wide meta‐analysis. Among them, rs17249754 was reported to have connection with SBP (Kato et al., 2011); rs17249754 and rs891151 with DBP (Kato et al., 2011; Liu et al., 2016); rs1173756, rs1173771, rs17477177, rs7437940 and rs4701131 with PP (Kelly et al., 2013; Kraja et al., 2017; Surendran et al., 2016; Wain et al., 2011); rs1173771, rs2681472, rs2681492, rs17249754 and rs1004467 with MAP (Kelly et al., 2013; Wain et al., 2011). Some significant SNP were verified in our study, which added the credibility of our study.

#### 4.2.2 Imputation

Though no genome‐wide significant SNP was found after imputation, the number of available SNPs for GWAS analysis increased dramatically. That might provide more information for our study. SNP rs79259191 was located on the F‐box and leucine rich repeat protein 13 (*FBXL13*, OMIM accession number: 609080) which plays an important role in BP control and response (He et al., 2013). SNPs rs539006870 and rs13266333 were located on the LDL receptor related protein 1B (*LRP1B*, OMIM accession number: 608766) and *TRAPPC9* gene, respectively, which were found to have connection with BP regulation by exploiting gene‐smoking interactions from Framingham Heart Study (Sung et al., 2015). SNP rs10809095 was located on the protein tyrosine phosphatase receptor type D gene (*PTPRD*, OMIM accession number: 601598), which was associated with resistant hypertension in multiple ethnic groups (Gong et al., 2015), but the mechanism is still unclear. SNP rs4483351 was located on PR/SET domain 16 gene (*PRDM16*, OMIM accession number: 605557), which was found to have connection with cardiomyopathy (Arndt & MacRae, 2014; Arndt et al., 2013), thus it might could play an important role in regulating blood pressure.

#### 4.2.3 Gene‐based analysis

Zinc finger protein 580 (*ZNF580*, OMIM accession number: 617888) could regulate endothelial nitric oxide synthase (eNOS) expression via transforming growth factor‐β1 (*TGF*‐*β1*) pathway (Luo et al., 2014), and eNOS plays an important role in promoting vascular endothelial cell repair and maintaining normal cardiovascular diastolic function (Huang, 2009). So, *ZNF580* could regulate BP and have influences on some cardiovascular diseases, such as hypertension, atherosclerosis and so on. Furthermore, a study (DangLi et al., 2012) conducted by Ren et al. revealed that *ZNF580* could mediate vascular endothelial inflammation response by elevating cytokine IL‐8 expression, which played an important role in regulating BP.

S100 calcium binding protein A9 (*S100A9*, OMIM accession number: 123886) in atherosclerotic plaque could influence redox and Ca^2+^‐dependent processes, which might cause dystrophic calcification (McCormick et al., 2005). So, systolic and diastolic function of vascular is affected and blood pressure could also be affected. A study conducted by Eggers et al. (Eggers et al., 2011) indicated the release of *S100A9* could lead to increased cardiovascular risk and another study (Volz et al., 2012) showed that *S100A9* knockdown could cause reduced cellular proliferation, neointimal formation and atherosclerosis. These evidences indicated a modulatory role of the *S100A9* in vascular inflammation.

Epidermal growth factor receptor (*EGFR*, OMIM accession number: 131550) could recruit transient receptor potential classical type 6 (*TRPC6*) and transient receptor potential melastatin type 4 *(TRPM4*) channels, lastly stimulating voltage‐dependent calcium channels and potentiating myogenic tone (Carnevale et al., 2018). So, *EGFR* plays an important role in regulating BP. Previous studies have indicated that activation of *EGFR* is related to BP regulation, endothelial dysfunction, neointimal hyperplasia, atherogenesis, and cardiac remodeling (Makki et al., 2013; Schreier et al., 2014).

We further compared our results with some previous genome‐wide meta‐analysis (Bhatnagar et al., 2013; Huan et al., 2015; Kato et al., 2011; Kelly et al., 2013; C. Li, Zhang, et al., 2017; Surendran et al., 2016; Wain et al., 2011). Some BP‐related genes founded in our GWAS study had been reported in previous meta‐analysis, such as *THRB*, pleckstrin homology and RhoGEF domain containing G1 (*PLEKHG1*, OMIM accession number: NA), WW domain‐binding protein 1 like (*WBP1L*, OMIM accession number: 611129), sideroflexin 2 (*SFXN2*, OMIM accession number: 615570), arsenite methyltransferase (*AS3MT*, OMIM accession number: 611806), granulysin (*GNLY*, OMIM accession number: 188855), AHNAK nucleoprotein (*AHNAK*, OMIM accession number: 103390), microtubule associated protein 6 (*MAP6*, OMIM accession number: 601783), ATPase plasma membrane Ca2+ transporting 1 (*ATP2B1*, OMIM accession number: 108731), SUFU negative regulator of hedgehog signaling (*SUFU*, OMIM accession number: 607035), adhesion G protein‐coupled receptor E5 (*ADGRE5* or *CD97*, OMIM accession number: 601211), ABO, alpha 1‐3‐N‐acetylgalactosaminyltransferase and alpha 1‐3‐galactosyltransferase (*ABO*, OMIM accession number: 110300), ADP ribosylation factor like GTPase 3 (*ARL3*, OMIM accession number: 604695), actin related protein 1A (*ACTR1A*, OMIM accession number: 605143) and so on. These evidences also provide powerful support for our study.

#### 4.2.4 Pathway enrichment analysis

##### SBP

Several biological pathways were found to have significant associations with SBP: dilated cardiomyopathy (DCM), hormone ligand‐binding receptors, EGFR smrte pathway, and tyrosine metabolism. Apart from the top 20 pathways, other pathways might also have biological association with SBP. More studies need to be conducted to verify these associations.

(1) DCM is characterized by increased myocardial mass and volume, which could be caused by inflammation, autoimmunity and other factors (Luk et al., 2009; Zhao et al., 2009). Due to dysfunction of myocardium, the role of heart in regulating BP could be affected. So, normal BP would be affected. (2) Hormone ligand‐binding receptors could influence the combination of hormone ligand and class A (rhodopsin‐like) GPCRs, which could mediate the release of follicle‐stimulating hormone, luteinizing hormone and so on. And further affect the release of thyroid hormone, which plays an important role in regulating myocardium and BP. (3) EGFR smrte pathway participates the regulation of EGFR. The role of EGFR in affecting BP have been discussed in our study (Carnevale et al., 2018). (4) Tyrosine metabolism could influence catecholamine biosynthesis (tyrosine, dopamine, noradrenaline, adrenaline). The role of adrenaline in regulating BP is already well known.

##### DBP

Several biological pathways were found to have significant association with DBP: GAB1 signalosome, EGFR downregulation, SHC1 events in EGFR signaling, and EGFR smrte pathway.

GAB1 is recruited to the activated *EGFR* through GRB2, and EGFR downregulation, SHC1 events in EGFR signaling, and EGFR smrte pathway could interact with *EGFR* directly or indirectly, thus affect the downstream signals of EGFR.

##### PP

Several biological pathways were found to have significant associations with PP: platelet aggregation plug formation, integrin alphaiib beta3 signaling, tyrosine metabolism, and EGFR smrte pathway.

(1) Platelet aggregation plug formation is crucial for normal hemostasis, but pathological thrombus formation could also cause serious cardiovascular diseases such as stroke and atherosclerosis (Ruggeri & Mendolicchio, 2007; Varga‐Szabo et al., 2008). (2) Integrin alphaiib beta3 signaling could also participate the process of platelet activation and thrombosis (Parise, 1999; Shattil, 1999). So, intravascular hemodynamics and BP could be affected.

##### MAP

Several biological pathways were found to have significant associations with MAP: GNRH signaling pathway, signal transduction by L1, EGFR downregulation, SHC1 events in EGFR signaling.

(1) GNRH receptor could be coupled with G‐proteins, which mediate a wide variety of pathologies, such as cardiovascular, inflammatory and other diseases (Naor, 2009). (2) Signal transduction by L1 could interact with FGF receptor and activate DAG, resulting in the production of arachidonic acid, which plays an important role in BP regulation and hypertension (Capdevila et al., 2007; Kirkebo et al., 2000).

### 4.3 Strengths and limitations

Several advantages exist in our study. First, the results and conclusions of this study were based on Qingdao twin population, which increased the power of genetic analysis of BP (Tan et al., 2017). Second, to our knowledge, the number of GWAS investigating SBP, DBP, PP, and MAP among Asian simultaneously is relatively small, thus our study might provide some evidences for further investigations. Third, we discussed the genetic variation of blood pressure from different levels such as SNPs, genes, and pathways. Nevertheless, some potential limitations also exist in our study. First, because of the difficulties of collecting and identifying qualified twin pairs, sample size of this GWAS was relatively small, which might decrease the power of analysis. So, further studies need to be conducted to confirm our results. Second, due to the limitation of sample size, we did not perform gender stratification to observe the genetic differences between male and female. However, previous studies (Hottenga et al., 2005; Scurrah et al., 2006; Snieder et al., 2003; Wang et al., 2011) have revealed that there was no difference in blood pressure heritability between different sexes. Third, none genes reached the genome‐wide significance level in our study, but many genes were nominally associated with the blood pressure level (*p* < 0.05), some of which had been confirmed to have a biological connection with blood pressure.

## 5 CONCLUSION

In brief, SBP, DBP, PP, and MAP levels are moderately heritable in the Chinese population. BP could be mediate by a series of genomic loci, functional genes and biopathways and some related SNPs, genes and biopathways were found in our study. However, further large‐scale studies are needed to confirm our findings.

## CONFLICT OF INTEREST

No conflict of interest and no competing financial interest exist in the submission of this article.

## AUTHOR CONTRIBUTIONS

JC and DZ designed the study. CX and XT collected samples and phenotypes. WW and ZL assisted in sample data and sequencing data management. JC and WW analyzed the sequencing data and interpreted the analysis results. JC and WW drafted the manuscript, ZL and XT participated in the discussion, and CX, and DZ revised it. All the authors read the manuscript and agreed to publish. All the authors agreed to be responsible for all aspects of the work.

## Supporting information

Table S1

Table S2

Table S3

Table S4

Table S5

Table S6

Table S7

Table S8

Table S9

Table S10

Table S11

Table S12

Table S13
