# Supplementary material for: Heritability and genome‐wide association study of blood pressure in Chinese adult twins
Source: Mol Genet Genomic Med. 2021 Sep 29;9(11):e1828. doi: 10.1002/mgg3.1828 (PMC8606211; doi:10.1002/mgg3.1828)
Supplement: Supplementary file 1 — Table S1 [file MGG3-9-e1828-s005.doc]

| **Supplemental Table 1** The summary of SNPs with P-value < 1 × 10^-5^ for association with SBP in imputed genome-wide association study | | | | | |
| --- | --- | --- | --- | --- | --- |
| SNP | CHR | BP | P-value | Closest genes or genes | Official full name |
| rs58113664 | 2 | 43317460 | 5.58E-08 | RNU6-242P | RNA, U6 small nuclear 242, pseudogene |
| rs7574283 | 2 | 43314287 | 5.78E-08 | RNU6-242P | RNA, U6 small nuclear 242, pseudogene |
| [rs540063109](https://www.ncbi.nlm.nih.gov/snp/rs540063109) | 7 | 19534588 | 1.31E-07 | LOC105375180 | Uncharacterized |
| rs4952963 | 2 | 43319155 | 1.65E-07 | RNU6-242P | RNA, U6 small nuclear 242, pseudogene |
| rs72733628 | 14 | 77085230 | 2.59E-07 | CYCSP1 | CYCS pseudogene 1 |
| rs199906663 | 5 | 63848998 | 3.39E-07 | RGS7BP | Regulator of G protein signaling 7 binding protein |
| rs45563638 | 2 | 43314000 | 3.92E-07 | RNU6-242P | RNA, U6 small nuclear 242, pseudogene |
| rs10009930 | 4 | 54454564 | 6.34E-07 | LNX1 | Ligand of numb-protein X 1 |
| rs376742174 | 10 | 6166143 | 9.85E-07 | LOC101928080 | Uncharacterized |
| rs74934234 | 10 | 6173391 | 2.17E-06 | LOC101928080 | Uncharacterized |
| rs78151823 | 10 | 6170662 | 2.17E-06 | LOC101928080 | Uncharacterized |
| rs79723697 | 10 | 6172400 | 2.17E-06 | LOC101928080 | Uncharacterized |
| rs79868704 | 10 | 6172389 | 2.17E-06 | LOC101928080 | Uncharacterized |
| rs75814559 | 10 | 6180100 | 2.17E-06 | LOC101928080 | Uncharacterized |
| rs76323704 | 10 | 6180873 | 2.17E-06 | LOC101928080 | Uncharacterized |
| rs76372706 | 10 | 6177622 | 2.17E-06 | LOC101928080 | Uncharacterized |
| rs141898634 | 10 | 6160416 | 2.23E-06 | RBM17 | RNA binding motif protein 17 |
| rs76367377 | 10 | 6158989 | 2.23E-06 | RBM17 | RNA binding motif protein 17 |
| rs79980908 | 10 | 6161494 | 2.23E-06 | LOC101928080 | Uncharacterized |
| rs79820364 | 10 | 6168175 | 2.60E-06 | LOC101928080 | Uncharacterized |
| rs77793921 | 10 | 6186121 | 3.06E-06 | PFKFB3 | 6-phosphofructo-2-kinase/fructose-2,6-biphosphatase 3 |
| rs146444983 | 10 | 6164714 | 3.13E-06 | LOC101928080 | Uncharacterized |
| rs77294181 | 10 | 6163933 | 3.13E-06 | LOC101928080 | Uncharacterized |
| rs78782176 | 10 | 6164080 | 3.13E-06 | LOC101928080 | Uncharacterized |
| rs78876784 | 10 | 6164646 | 3.13E-06 | LOC101928080 | Uncharacterized |
| rs149777838 | 5 | 67672452 | 3.78E-06 | LOC105379013 | Uncharacterized |
| rs34710727 | 1 | 146997592 | 4.28E-06 | LINC00624 | Long intergenic non-protein coding RNA 624 |
| [rs866118481](https://www.ncbi.nlm.nih.gov/snp/rs866118481) | 4 | 5724918 | 4.31E-06 | EVC | EvC ciliary complex subunit 1 |
| rs965835 | 2 | 234748814 | 4.33E-06 | HJURP | Holliday junction recognition protein |
| rs35107828 | 1 | 146996947 | 4.98E-06 | LINC00624 | Long intergenic non-protein coding RNA 624 |
| rs34400696 | 1 | 146997181 | 4.98E-06 | LINC00624 | Long intergenic non-protein coding RNA 624 |
| rs965498542 | 16 | 52262956 | 5.36E-06 | LOC105371261 | Uncharacterized |
| rs539006870 | 2 | 142518974 | 5.52E-06 | LRP1B | LDL receptor related protein 1B |
|  |  |  |  | LOC107985779 | Uncharacterized |
| rs141761366 | 12 | 26441808 | 6.16E-06 | LOC105369705 | Uncharacterized |
| rs183441903 | 1 | 150236264 | 6.44E-06 | CA14 | Carbonic anhydrase 14 |
| rs56963308 | 2 | 43320765 | 6.56E-06 | RNU6-242P | RNA, U6 small nuclear 242, pseudogene |
| rs79259191 | 7 | 102674954 | 6.59E-06 | FBXL13 | F-box and leucine rich repeat protein 13 |
| rs79406484 | 10 | 6143955 | 6.69E-06 | RBM17 | RNA binding motif protein 17 |
| rs75256238 | 10 | 6147226 | 6.69E-06 | RBM17 | RNA binding motif protein 17 |
| rs1355870272 | 15 | 38888773 | 7.17E-06 | LOC107984725 | Uncharacterized |
| rs376804862 | 3 | 93958905 | 7.26E-06 | NSUN3 | NOP2/Sun RNA methyltransferase 3 |
| rs59656854 | 9 | 18019692 | 9.42E-06 | ADAMTSL1 | ADAMTS like 1 |
| rs34438904 | 15 | 81400681 | 9.67E-06 | CFAP161 | Cilia and flagella associated protein 161 |
| rs10787239 | 10 | 112220829 | 9.85E-06 | HMGB3P5 | High mobility group box 3 pseudogene 5 |
| rs11256258 | 10 | 6033415 | 9.93E-06 | IL15RA | Interleukin 15 receptor subunit alpha |
| rs4952964 | 2 | 43323393 | 9.94E-06 | LINC02580 | Long intergenic non-protein coding RNA 2580 |
| SBP, systolic blood pressure; CHR, chromosome; BP, base pair  SNPs information was from Build 38 (GRCh38) | | | | | |
