# Supplementary material for: Heritability and genome‐wide association study of blood pressure in Chinese adult twins
Source: Mol Genet Genomic Med. 2021 Sep 29;9(11):e1828. doi: 10.1002/mgg3.1828 (PMC8606211; doi:10.1002/mgg3.1828)
Supplement: Supplementary file 2 — Table S2 [file MGG3-9-e1828-s010.doc]

| **Supplemental Table 2** The summary of SNPs with P-value < 1 × 10^-5^ for association with imputed DBP in genome-wide association study | | | | | |
| --- | --- | --- | --- | --- | --- |
| SNP | CHR | BP | P-value | Closest genes or genes | Official full name |
| rs141669870 | 14 | 86096147 | 1.77E-06 | FLRT2 | Fibronectin leucine rich transmembrane protein 2 |
| rs4599441 | 4 | 63227981 | 3.46E-06 | LOC100289193 | Uncharacterized |
| rs1489617829 | 12 | 69465786 | 3.50E-06 | LOC107984471 | Uncharacterized |
| rs6734703 | 2 | 153798065 | 3.86E-06 | LOC105373691 | Uncharacterized |
| rs79259191 | 7 | 102674954 | 4.02E-06 | FBXL13 | F-box and leucine rich repeat protein 13 |
| rs1524045 | 2 | 153790316 | 4.30E-06 | LOC105373691 | Uncharacterized |
| rs972541 | 2 | 153791428 | 4.59E-06 | LOC105373691 | Uncharacterized |
| rs10637618 | 5 | 79657118 | 4.74E-06 | HNRNPA1P12 | Heterogeneous nuclear ribonucleoprotein A1 pseudogene 12 |
| rs6730158 | 2 | 153789409 | 4.80E-06 | LOC105373691 | Uncharacterized |
| rs34941231 | 2 | 153797326 | 4.84E-06 | LOC105373691 | Uncharacterized |
| rs13374999 | 1 | 181074884 | 5.55E-06 | IER5 | Immediate early response 5 |
| rs67376518 | 1 | 181068916 | 5.76E-06 | IER5 | Immediate early response 5 |
| rs56245736 | 6 | 86978139 | 5.93E-06 | NDUFA5P9 | NADH:ubiquinone oxidoreductase subunit A5 pseudogene 9 |
| rs72905299 | 6 | 86970406 | 5.93E-06 | NDUFA5P9 | NADH:ubiquinone oxidoreductase subunit A5 pseudogene 9 |
| rs72907104 | 6 | 86971503 | 5.93E-06 | NDUFA5P9 | NADH:ubiquinone oxidoreductase subunit A5 pseudogene 9 |
| rs146132795 | 6 | 86979672 | 5.93E-06 | NDUFA5P9 | NADH:ubiquinone oxidoreductase subunit A5 pseudogene 9 |
| rs1936179 | 6 | 86984149 | 5.93E-06 | NDUFA5P9 | NADH:ubiquinone oxidoreductase subunit A5 pseudogene 9 |
| rs140632662 | 6 | 86994320 | 5.93E-06 | NDUFA5P9 | NADH:ubiquinone oxidoreductase subunit A5 pseudogene 9 |
| rs138417242 | 6 | 49500281 | 6.30E-06 | GLYATL3 | Glycine-N-acyltransferase like 3 |
| rs57037058 | 14 | 82437952 | 6.59E-06 | EIF3LP1 | Eukaryotic translation initiation factor 3 subunit L pseudogene 1 |
| rs377214741 | 2 | 153785815 | 6.83E-06 | LOC105373691 | Uncharacterized |
| rs13003360 | 2 | 153786255 | 6.83E-06 | LOC105373691 | Uncharacterized |
| rs111208124 | 2 | 153785888 | 6.83E-06 | LOC105373691 | Uncharacterized |
| rs1916808 | 2 | 153786358 | 6.83E-06 | LOC105373691 | Uncharacterized |
| rs66675954 | 2 | 153786388 | 6.83E-06 | UBQLN4P2 | Ubiquilin 4 pseudogene 2 |
| rs4456652 | 2 | 153757506 | 7.38E-06 | UBQLN4P2 | Ubiquilin 4 pseudogene 2 |
| rs12990814 | 2 | 153761995 | 7.38E-06 | UBQLN4P2 | Ubiquilin 4 pseudogene 2 |
| rs34081175 | 2 | 153767583 | 7.38E-06 | UBQLN4P2 | Ubiquilin 4 pseudogene 2 |
| rs34326233 | 2 | 153770846 | 7.38E-06 | UBQLN4P2 | Ubiquilin 4 pseudogene 2 |
| rs79110951 | 11 | 7904064 | 7.46E-06 | LOC283299 | Uncharacterized |
| rs78970479 | 11 | 90008402 | 8.00E-06 | DISC1FP1 | DISC1 fusion partner 1 |
| rs72695476 | 14 | 82429884 | 8.60E-06 | EIF3LP1 | Eukaryotic translation initiation factor 3 subunit L pseudogene 1 |
| rs1225001170 | 19 | 20125423 | 8.77E-06 | ZNF682 | Zinc finger protein 682 |
| rs7127355 | 11 | 80045351 | 9.31E-06 | LOC105369408 | Uncharacterized |
| rs201222654 | 2 | 153796194 | 9.49E-06 | LOC105373691 | Uncharacterized |
| rs1395594 | 16 | 7995283 | 9.58E-06 | LOC105371070 | Uncharacterized |
| rs138031614 | 1 | 166610027 | 9.83E-06 | FMO10P | Flavin containing dimethylaniline monoxygenase 10, pseudogene |
| DBP, diastolic blood pressure; CHR, chromosome; BP, base pair  SNPs information was from Build 38 (GRCh38) | | | | | |
