# Supplementary material for: Heritability and genome‐wide association study of blood pressure in Chinese adult twins
Source: Mol Genet Genomic Med. 2021 Sep 29;9(11):e1828. doi: 10.1002/mgg3.1828 (PMC8606211; doi:10.1002/mgg3.1828)
Supplement: Supplementary file 3 — Table S3 [file MGG3-9-e1828-s003.doc]

| **Supplemental Table 3** The summary of SNPs with P-value < 1 × 10^-5^ for association with PP in imputed genome-wide association study | | | | | |
| --- | --- | --- | --- | --- | --- |
| SNP | CHR | BP | P-value | Closest genes or genes | Official full name |
| rs148306575 | 5 | 160988537 | 1.30E-07 | GABRB2 | Gamma-aminobutyric acid type A receptor beta2 subunit |
| rs57731910 | 15 | 38924503 | 1.62E-07 | LOC107984725 | Uncharacterized |
| rs11353405 | 15 | 38923685 | 1.99E-07 | LOC107984725 | Uncharacterized |
| rs56207680 | 5 | 160988697 | 6.03E-07 | GABRB2 | Gamma-aminobutyric acid type A receptor beta2 subunit |
| rs72815554 | 5 | 160995760 | 6.03E-07 | GABRB2 | Gamma-aminobutyric acid type A receptor beta2 subunit |
| rs72815526 | 5 | 160976104 | 6.20E-07 | GABRB2 | Gamma-aminobutyric acid type A receptor beta2 subunit |
| rs56234890 | 5 | 160979720 | 7.63E-07 | GABRB2 | Gamma-aminobutyric acid type A receptor beta2 subunit |
| rs12583722 | 13 | 84695187 | 1.14E-06 | LINC00333 | Long intergenic non-protein coding RNA 333 |
| rs45563638 | 2 | 43314000 | 1.56E-06 | RNU6-242P | RNA, U6 small nuclear 242, pseudogene |
| rs12153198 | 5 | 160915124 | 1.66E-06 | GABRB2 | Gamma-aminobutyric acid type A receptor beta2 subunit |
| rs6881515 | 5 | 160911968 | 1.66E-06 | GABRB2 | Gamma-aminobutyric acid type A receptor beta2 subunit |
| rs9551857 | 13 | 30724073 | 1.83E-06 | LINC00384 | Long intergenic non-protein coding RNA 384 |
| rs4341632 | 13 | 97081244 | 2.08E-06 | HS6ST3 | Heparan sulfate 6-O-sulfotransferase 3 |
| rs757361398 | 23 | 92601573 | 2.22E-06 | LOC100131340 | Uncharacterized |
| rs77199255 | 15 | 38916982 | 2.31E-06 | LOC109784725 | Uncharacterized |
| rs138312965 | 5 | 160960111 | 2.40E-06 | GABRB2 | Gamma-aminobutyric acid type A receptor beta2 subunit |
| rs72815551 | 5 | 160995192 | 2.54E-06 | GABRB2 | Gamma-aminobutyric acid type A receptor beta2 subunit |
| rs58113664 | 2 | 43317460 | 2.60E-06 | RNU6-242P | RNA, U6 small nuclear 242, pseudogene |
| rs11135150 | 5 | 160915001 | 2.61E-06 | GABRB2 | Gamma-aminobutyric acid type A receptor beta2 subunit |
| rs12153421 | 5 | 160917585 | 2.61E-06 | GABRB2 | Gamma-aminobutyric acid type A receptor beta2 subunit |
| rs56114853 | 5 | 160914114 | 2.61E-06 | GABRB2 | Gamma-aminobutyric acid type A receptor beta2 subunit |
| rs62381574 | 5 | 160918447 | 2.61E-06 | GABRB2 | Gamma-aminobutyric acid type A receptor beta2 subunit |
| rs72813581 | 5 | 160913479 | 2.61E-06 | GABRB2 | Gamma-aminobutyric acid type A receptor beta2 subunit |
| rs12153038 | 5 | 160921475 | 2.61E-06 | GABRB2 | Gamma-aminobutyric acid type A receptor beta2 subunit |
| rs12189094 | 5 | 160923428 | 2.61E-06 | GABRB2 | Gamma-aminobutyric acid type A receptor beta2 subunit |
| rs72813589 | 5 | 160924163 | 2.61E-06 | GABRB2 | Gamma-aminobutyric acid type A receptor beta2 subunit |
| rs142435015 | 5 | 160939026 | 2.61E-06 | GABRB2 | Gamma-aminobutyric acid type A receptor beta2 subunit |
| rs62381577 | 5 | 160931928 | 2.61E-06 | GABRB2 | Gamma-aminobutyric acid type A receptor beta2 subunit |
| rs62381580 | 5 | 160937349 | 2.61E-06 | GABRB2 | Gamma-aminobutyric acid type A receptor beta2 subunit |
| rs72813594 | 5 | 160930142 | 2.61E-06 | GABRB2 | Gamma-aminobutyric acid type A receptor beta2 subunit |
| rs72813596 | 5 | 160931729 | 2.61E-06 | GABRB2 | Gamma-aminobutyric acid type A receptor beta2 subunit |
| rs17522604 | 5 | 160942725 | 2.61E-06 | GABRB2 | Gamma-aminobutyric acid type A receptor beta2 subunit |
| rs57248301 | 5 | 160939996 | 2.61E-06 | GABRB2 | Gamma-aminobutyric acid type A receptor beta2 subunit |
| rs61647897 | 5 | 160940596 | 2.61E-06 | GABRB2 | Gamma-aminobutyric acid type A receptor beta2 subunit |
| rs62381583 | 5 | 160941481 | 2.61E-06 | GABRB2 | Gamma-aminobutyric acid type A receptor beta2 subunit |
| rs17463127 | 5 | 160952502 | 2.61E-06 | GABRB2 | Gamma-aminobutyric acid type A receptor beta2 subunit |
| rs73384202 | 15 | 38892085 | 2.66E-06 | LOC107984725 | Uncharacterized |
| rs11956795 | 5 | 160983125 | 2.68E-06 | GABRB2 | Gamma-aminobutyric acid type A receptor beta2 subunit |
| rs1355870272 | 15 | 38888773 | 2.71E-06 | LOC107984725 | Uncharacterized |
| rs10152478 | 15 | 38887698 | 2.84E-06 | LOC107984739 | Uncharacterized |
| rs4924278 | 15 | 38886440 | 3.07E-06 | LOC107984739 | Uncharacterized |
| rs7174144 | 15 | 38910797 | 3.33E-06 | LOC107984725 | Uncharacterized |
| rs7173588 | 15 | 38910544 | 3.55E-06 | LOC107984725 | Uncharacterized |
| rs883625 | 15 | 38918032 | 3.91E-06 | LOC107984725 | Uncharacterized |
| rs1316583 | 15 | 38886046 | 4.10E-06 | LOC107984739 | Uncharacterized |
| rs73384196 | 15 | 38885659 | 4.10E-06 | LOC107984739 | Uncharacterized |
| rs72815522 | 5 | 160962283 | 4.13E-06 | GABRB2 | Gamma-aminobutyric acid type A receptor beta2 subunit |
| rs61796821 | 3 | 158581729 | 4.24E-06 | LOC105374181 | Uncharacterized |
| rs9875783 | 3 | 82477805 | 4.35E-06 | LINC02008 | Long intergenic non-protein coding RNA 2008 |
| rs10152667 | 15 | 38885114 | 4.41E-06 | LOC107984739 | Uncharacterized |
| rs58128961 | 15 | 38914152 | 4.61E-06 | LOC107984725 | Uncharacterized |
| rs8024875 | 15 | 38905784 | 4.71E-06 | LOC107984725 | Uncharacterized |
| rs1261043632 | 2 | 183773058 | 4.87E-06 | NCKAP1 | NCK associated protein 1 |
| rs7164418 | 15 | 38912822 | 5.36E-06 | LOC107984725 | Uncharacterized |
| rs4923802 | 15 | 38881245 | 5.90E-06 | LOC107984739 | Uncharacterized |
| rs61796848 | 3 | 158589967 | 5.94E-06 | LOC105374181 | Uncharacterized |
| rs2319751 | 15 | 38885865 | 6.25E-06 | LOC107984739 | Uncharacterized |
| rs12548023 | 8 | 140915692 | 6.44E-06 | TRAPPC9 | Trafficking protein particle complex 9 |
| rs10096902 | 8 | 140914837 | 6.72E-06 | TRAPPC9 | Trafficking protein particle complex 9 |
| rs10097473 | 8 | 140914630 | 6.72E-06 | TRAPPC9 | Trafficking protein particle complex 9 |
| rs10107700 | 8 | 140915017 | 6.72E-06 | TRAPPC9 | Trafficking protein particle complex 9 |
| rs12547470 | 8 | 140915519 | 6.72E-06 | TRAPPC9 | Trafficking protein particle complex 9 |
| rs12547471 | 8 | 140915520 | 6.72E-06 | TRAPPC9 | Trafficking protein particle complex 9 |
| rs28556916 | 8 | 140915442 | 6.72E-06 | TRAPPC9 | Trafficking protein particle complex 9 |
| rs6982104 | 8 | 140912215 | 6.74E-06 | TRAPPC9 | Trafficking protein particle complex 9 |
| rs1075493 | 8 | 140917457 | 7.01E-06 | TRAPPC9 | Trafficking protein particle complex 9 |
| rs67701708 | 8 | 140916796 | 7.01E-06 | TRAPPC9 | Trafficking protein particle complex 9 |
| rs10809095 | 9 | 10468784 | 7.09E-06 | PTPRD | Protein tyrosine phosphatase receptor type D |
| rs79664160 | 3 | 130415306 | 7.14E-06 | PIK3R4 | Phosphoinositide-3-kinase regulatory subunit 4 |
| rs11174608 | 12 | 40380583 | 7.22E-06 | SLC2A13 | Solute carrier family 2 member 13 |
| rs75471160 | 13 | 107457289 | 7.33E-06 | LOC105370349 | Uncharacterized |
| rs7364440 | 1 | 200908902 | 7.58E-06 | MROH3P | Maestro heat like repeat family member 3, pseudogene |
| rs13340349 | 5 | 73429725 | 7.62E-06 | LINC02122 | Long intergenic non-protein coding RNA 2122 |
| rs10584210 | 18 | 41039774 | 7.69E-06 | SYT4 | Synaptotagmin 4 |
| rs13266333 | 8 | 140913144 | 7.90E-06 | TRAPPC9 | Trafficking protein particle complex 9 |
| rs1007203 | 15 | 38880060 | 7.98E-06 | LOC107984739 | Uncharacterized |
| rs16967039 | 15 | 38880420 | 7.98E-06 | LOC107984739 | Uncharacterized |
| rs76945286 | 15 | 38908264 | 8.03E-06 | LOC107984725 | Uncharacterized |
| rs35440803 | 1 | 236620413 | 8.08E-06 | EDARADD | EDAR associated death domain |
| rs375433031 | 5 | 177696288 | 8.46E-06 | COL23A1 | Collagen type XXIII alpha 1 chain |
| rs140618707 | 10 | 121287896 | 8.79E-06 | RGS10 | Regulator of G protein signaling 10 |
| rs7175048 | 15 | 38882930 | 8.80E-06 | LOC107984739 | Uncharacterized |
| rs9550532 | 13 | 30723602 | 8.91E-06 | LINC00384 | Long intergenic non-protein coding RNA 384 |
| rs913905 | 13 | 30720425 | 8.93E-06 | LINC00384 | Long intergenic non-protein coding RNA 384 |
| rs4483351 | 1 | 3117444 | 8.97E-06 | PRDM16 | PR/SET domain 16 |
| rs4915466 | 1 | 200905507 | 9.02E-06 | MROH3P | Maestro heat like repeat family member 3, pseudogene |
| rs11608410 | 12 | 105027342 | 9.05E-06 | CHST11 | Carbohydrate sulfotransferase 11 |
| rs1554075 | 4 | 138119507 | 9.30E-06 | LINC02510 | Long intergenic non-protein coding RNA 2510 |
|  |  |  |  | LINC02511 | Long intergenic non-protein coding RNA 2511 |
| rs572043360 | 6 | 112951644 | 9.35E-06 | PA2G4P5 | Proliferation-associated 2G4 pseudogene 5 |
| rs146096598 | 11 | 132109586 | 9.41E-06 | NTM | Neurotrimin |
| rs4235077 | 4 | 86026327 | 9.95E-06 | RN7SKP48 | RN7SK pseudogene 48 |
| PP, pulse pressure; CHR, chromosome; BP, base pair  SNPs information was from Build 38 (GRCh38) | | | | | |
