# Supplementary material for: Heritability and genome‐wide association study of blood pressure in Chinese adult twins
Source: Mol Genet Genomic Med. 2021 Sep 29;9(11):e1828. doi: 10.1002/mgg3.1828 (PMC8606211; doi:10.1002/mgg3.1828)
Supplement: Supplementary file 4 — Table S4 [file MGG3-9-e1828-s011.doc]

| **Supplemental Table 4** The summary of SNPs with P-value < 1 × 10^-5^ for association with MAP in imputed genome-wide association study | | | | | |
| --- | --- | --- | --- | --- | --- |
| SNP | CHR | BP | P-value | Closest genes or genes | Official full name |
| rs79259191 | 7 | 102674954 | 3.66E-07 | FBXL13 | F-box and leucine rich repeat protein 13 |
| rs7822830 | 8 | 29394781 | 9.85E-07 | LOC105379350 | Uncharacterized |
| rs72733628 | 14 | 77085230 | 1.02E-06 | CYCSP1 | CYCS pseudogene 1 |
| rs77793921 | 10 | 6186121 | 1.03E-06 | PFKFB3 | 6-phosphofructo-2-kinase/fructose-2,6-biphosphatase 3 |
| rs376742174 | 10 | 6166143 | 1.18E-06 | LOC101928080 | Uncharacterized |
| rs74934234 | 10 | 6173391 | 1.22E-06 | LOC101928080 | Uncharacterized |
| rs78151823 | 10 | 6170662 | 1.22E-06 | LOC101928080 | Uncharacterized |
| rs79723697 | 10 | 6172400 | 1.22E-06 | LOC101928080 | Uncharacterized |
| rs79868704 | 10 | 6172389 | 1.22E-06 | LOC101928080 | Uncharacterized |
| rs75814559 | 10 | 6180100 | 1.22E-06 | LOC101928080 | Uncharacterized |
| rs76323704 | 10 | 6180873 | 1.22E-06 | LOC101928080 | Uncharacterized |
| rs76372706 | 10 | 6177622 | 1.22E-06 | LOC101928080 | Uncharacterized |
| rs79820364 | 10 | 6168175 | 1.92E-06 | LOC101928080 | Uncharacterized |
| rs146444983 | 10 | 6164714 | 1.94E-06 | LOC101928080 | Uncharacterized |
| rs77294181 | 10 | 6163933 | 1.94E-06 | LOC101928080 | Uncharacterized |
| rs78782176 | 10 | 6164080 | 1.94E-06 | LOC101928080 | Uncharacterized |
| rs78876784 | 10 | 6164646 | 1.94E-06 | LOC101928080 | Uncharacterized |
| rs7574283 | 2 | 43314287 | 2.00E-06 | RNU6-242P | RNA, U6 small nuclear 242, pseudogene |
| rs199906663 | 5 | 63848998 | 2.30E-06 | RGS7BP | Regulator of G protein signaling 7 binding protein |
| rs72695476 | 14 | 82429884 | 2.32E-06 | EIF3LP1 | Eukaryotic translation initiation factor 3 subunit L pseudogene 1 |
| rs141898634 | 10 | 6160416 | 2.56E-06 | RBM17 | RNA binding motif protein 17 |
| rs76367377 | 10 | 6158989 | 2.56E-06 | RBM17 | RNA binding motif protein 17 |
| rs79980908 | 10 | 6161494 | 2.56E-06 | RBM17 | RNA binding motif protein 17 |
| rs58113664 | 2 | 43317460 | 2.79E-06 | RNU6-242P | RNA, U6 small nuclear 242, pseudogene |
| rs6927364 | 6 | 12595275 | 3.11E-06 | LINC02530 | Long intergenic non-protein coding RNA 2530 |
| rs385753 | 4 | 39807565 | 3.30E-06 | RNA5SP159 | RNA, 5S ribosomal pseudogene 159 |
| rs57037058 | 14 | 82437952 | 3.60E-06 | EIF3LP1 | Eukaryotic translation initiation factor 3 subunit L pseudogene 1 |
| rs540063109 | 7 | 19534588 | 3.82E-06 | LOC105375180 | Uncharacterized |
| rs377214741 | 2 | 153785815 | 3.97E-06 | LOC105373691 | Uncharacterized |
| rs13003360 | 2 | 153786255 | 3.97E-06 | LOC105373691 | Uncharacterized |
| rs111208124 | 2 | 153785888 | 3.97E-06 | LOC105373691 | Uncharacterized |
| rs1916808 | 2 | 153786358 | 3.97E-06 | LOC105373691 | Uncharacterized |
| rs66675954 | 2 | 153786388 | 3.97E-06 | UBQLN4P2 | Ubiquilin 4 pseudogene 2 |
| rs4456652 | 2 | 153757506 | 3.99E-06 | UBQLN4P2 | Ubiquilin 4 pseudogene 2 |
| rs12990814 | 2 | 153761995 | 3.99E-06 | UBQLN4P2 | Ubiquilin 4 pseudogene 2 |
| rs34081175 | 2 | 153767583 | 3.99E-06 | UBQLN4P2 | Ubiquilin 4 pseudogene 2 |
| rs34326233 | 2 | 153770846 | 3.99E-06 | UBQLN4P2 | Ubiquilin 4 pseudogene 2 |
| rs4952963 | 2 | 43319155 | 4.57E-06 | RNU6-242P | RNA, U6 small nuclear 242, pseudogene |
| rs10009930 | 4 | 54454564 | 4.70E-06 | LNX1 | Ligand of numb-protein X 1 |
| rs72695477 | 14 | 82433929 | 5.81E-06 | EIF3LP1 | Eukaryotic translation initiation factor 3 subunit L pseudogene 1 |
| rs1888656 | 10 | 24833705 | 5.86E-06 | KIAA1217 | KIAA1217 |
| rs72830479 | 2 | 105450533 | 5.98E-06 | PANTR1 | POU3F3 adjacent non-coding transcript 1 |
| rs4717010 | 7 | 155981911 | 6.57E-06 | LOC105375601 | Uncharacterized |
| rs13244485 | 7 | 155982691 | 6.57E-06 | LOC105375601 | Uncharacterized |
| rs11648694 | 16 | 9118636 | 6.86E-06 | LOC105371074 | Uncharacterized |
| rs11762775 | 7 | 155987652 | 7.71E-06 | LOC105375601 | Uncharacterized |
| rs11763542 | 7 | 155987903 | 7.71E-06 | LOC105375601 | Uncharacterized |
| rs11645696 | 16 | 9118599 | 7.73E-06 | LOC105371074 | Uncharacterized |
| rs80250031 | 10 | 67033840 | 7.82E-06 | LOC100421870 | Uncharacterized |
| rs371471717 | 10 | 46254201 | 8.42E-06 | WASHC2C | WASH complex subunit 2C |
| rs57561750 | 10 | 67020070 | 8.56E-06 | LOC100421870 | Uncharacterized |
| rs79406484 | 10 | 6143955 | 8.61E-06 | RBM17 | RNA binding motif protein 17 |
| rs75256238 | 10 | 6147226 | 8.61E-06 | RBM17 | RNA binding motif protein 17 |
| rs319467 | 1 | 118290095 | 8.75E-06 | PNRC2P1 | Proline rich nuclear receptor coactivator 2 pseudogene 1 |
| rs11639706 | 16 | 9119249 | 8.75E-06 | LOC105371074 | Uncharacterized |
| rs12924501 | 16 | 9120946 | 8.75E-06 | LOC105371074 | Uncharacterized |
| rs12931602 | 16 | 9120958 | 8.75E-06 | LOC105371074 | Uncharacterized |
| rs12932565 | 16 | 9119155 | 8.75E-06 | LOC105371074 | Uncharacterized |
| rs72651595 | 1 | 27410176 | 8.78E-06 | LOC101928391 | Uncharacterized |
| rs72651596 | 1 | 27410274 | 8.78E-06 | SLC9A1 | Solute carrier family 9 member A1 |
| rs2504907 | 6 | 12603401 | 9.05E-06 | LINC02530 | Long intergenic non-protein coding RNA 2530 |
| MAP, mean arterial pressure; CHR, chromosome; BP, base pair  SNPs information was from Build 38 (GRCh38) | | | | | |
