# Supplementary material for: Heritability and genome‐wide association study of blood pressure in Chinese adult twins
Source: Mol Genet Genomic Med. 2021 Sep 29;9(11):e1828. doi: 10.1002/mgg3.1828 (PMC8606211; doi:10.1002/mgg3.1828)
Supplement: Supplementary file 5 — Table S5 [file MGG3-9-e1828-s007.doc]

| Supplemental Table 5 The top 20 genes from VEGAS2 gene-based analysis showing the strongest association with SBP level in typed GWAS data | | | | | | | | |
| --- | --- | --- | --- | --- | --- | --- | --- | --- |
| CHR | Gene | Numbers of SNPs | Start position | Stop position | Gene-based test statistic | Gene *P*-value | Top-SNP | Top-SNP *P*-value |
| 3 | THRB | 317 | 24158644 | 24536313 | 804.57 | 4.50E-05 | rs6777133 | 3.15E-05 |
| 17 | PSMB3 | 5 | 36908965 | 36920484 | 36.98 | 1.03E-04 | rs668461 | 3.64E-04 |
| 19 | ZNF576 | 5 | 44100543 | 44104587 | 24.72 | 1.42E-04 | rs2240932 | 1.46E-03 |
| 11 | OR8D1 | 4 | 124179735 | 124180662 | 29.45 | 2.23E-04 | rs4936919 | 1.49E-03 |
| 6 | SLC35B2 | 5 | 44221837 | 44225627 | 27.21 | 2.24E-04 | rs3734707 | 1.02E-03 |
| 14 | IPO4 | 9 | 24649424 | 24658124 | 56.60 | 2.33E-04 | rs7146310 | 3.99E-04 |
| 17 | LOC102724596 | 8 | 47438526 | 47457456 | 61.98 | 2.35E-04 | rs16948048 | 3.05E-04 |
| 15 | BCL2A1 | 8 | 80253231 | 80263643 | 60.38 | 2.42E-04 | rs6495460 | 2.76E-04 |
| 12 | ORMDL2 | 2 | 56211805 | 56214959 | 16.53 | 3.15E-04 | rs56108400 | 2.68E-03 |
| 1 | ANGPTL7 | 9 | 11249345 | 11256038 | 41.74 | 3.17E-04 | rs28990992 | 4.83E-04 |
| 1 | CD1A | 7 | 158223926 | 158228058 | 46.92 | 3.61E-04 | rs2269715 | 1.28E-03 |
| 19 | ZNF682 | 21 | 20115226 | 20150277 | 113.54 | 4.36E-04 | rs11085292 | 4.63E-04 |
| 19 | ZNF580 | 3 | 56152391 | 56154836 | 21.32 | 4.74E-04 | rs310475 | 2.18E-04 |
| 2 | GTF2A1L | 32 | 48844918 | 48906751 | 260.92 | 4.90E-04 | rs34003293 | 1.43E-04 |
| 19 | MBD3 | 4 | 1576669 | 1592760 | 29.35 | 4.90E-04 | rs4807934 | 6.01E-04 |
| 11 | KCNK7 | 5 | 65360325 | 65363467 | 29.19 | 4.95E-04 | rs76353556 | 2.51E-03 |
| 7 | GHRHR | 21 | 31003635 | 31019146 | 93.18 | 5.19E-04 | rs2074781 | 8.21E-04 |
| 17 | TMEM220 | 18 | 10616638 | 10633646 | 139.44 | 5.45E-04 | rs368500 | 1.38E-04 |
| 19 | RINL | 9 | 39358471 | 39368919 | 50.47 | 5.52E-04 | rs10424892 | 2.53E-04 |
| 11 | SLC37A4 | 7 | 118895060 | 118901616 | 39.48 | 5.56E-04 | rs11006 | 3.40E-04 |
| SBP, systolic blood pressure; CHR, chromosome. | | | | | | | | |
