# Supplementary material for: Heritability and genome‐wide association study of blood pressure in Chinese adult twins
Source: Mol Genet Genomic Med. 2021 Sep 29;9(11):e1828. doi: 10.1002/mgg3.1828 (PMC8606211; doi:10.1002/mgg3.1828)
Supplement: Supplementary file 6 — Table S6 [file MGG3-9-e1828-s012.doc]

| Supplemental Table 6 The top 20 genes from VEGAS2 gene-based analysis showing the strongest association with DBP level in typed GWAS data | | | | | | | | |
| --- | --- | --- | --- | --- | --- | --- | --- | --- |
| CHR | Gene | Numbers of SNPs | Start position | Stop position | Gene-based test statistic | Gene *P*-value | Top-SNP | Top-SNP *P*-value |
| 1 | FMO9P | 8 | 166573152 | 166594473 | 98.90 | 1.80E-05 | rs7546913 | 1.65E-05 |
| 13 | LINC00346 | 6 | 111516333 | 111522655 | 59.90 | 2.90E-05 | rs9588287 | 5.66E-05 |
| 2 | FEV | 2 | 219845808 | 219850379 | 32.56 | 4.60E-05 | rs860573 | 5.21E-05 |
| 21 | TFF2 | 3 | 43766466 | 43771208 | 37.35 | 5.90E-05 | rs751143 | 5.95E-05 |
| 1 | S100A9 | 2 | 153330329 | 153333503 | 18.57 | 2.10E-04 | rs11544410 | 2.70E-05 |
| 11 | SLC37A4 | 7 | 118895060 | 118901616 | 43.89 | 2.32E-04 | rs11006 | 1.30E-04 |
| 3 | ZNF619 | 5 | 40518603 | 40531728 | 27.30 | 2.66E-04 | rs9852518 | 7.46E-04 |
| 9 | COL5A1 | 190 | 137533650 | 137736688 | 598.27 | 2.77E-04 | rs7041099 | 4.60E-05 |
| 5 | SPZ1 | 5 | 79615789 | 79617660 | 26.32 | 2.96E-04 | rs1862136 | 2.01E-03 |
| 11 | TRAPPC4 | 3 | 118889240 | 118894385 | 32.89 | 3.29E-04 | rs569 | 1.30E-04 |
| 1 | NUCKS1 | 10 | 205681946 | 205719372 | 95.52 | 3.76E-04 | rs1772145 | 1.21E-04 |
| 11 | OR5M11 | 4 | 56309815 | 56310733 | 18.72 | 4.00E-04 | rs628524 | 1.88E-03 |
| 11 | OR5T2 | 7 | 55999581 | 56000661 | 41.29 | 4.54E-04 | rs12221615 | 6.09E-04 |
| 2 | LINC01158 | 14 | 105421882 | 105467934 | 81.75 | 4.80E-04 | rs4851716 | 4.79E-05 |
| 1 | ZSWIM5 | 39 | 45482075 | 45672250 | 253.64 | 4.92E-04 | rs2202152 | 3.89E-05 |
| 19 | MISP | 8 | 751145 | 764318 | 39.36 | 5.01E-04 | rs8112692 | 7.36E-04 |
| 14 | DTD2 | 8 | 31915242 | 31926680 | 49.42 | 5.07E-04 | rs8012658 | 1.22E-04 |
| 7 | EGFR | 110 | 55086724 | 55275031 | 383.50 | 5.08E-04 | rs13222385 | 1.00E-03 |
| 1 | LRRN2 | 44 | 204586302 | 204654597 | 183.84 | 6.90E-04 | rs41377246 | 8.92E-05 |
| 1 | FBLIM1 | 12 | 16085254 | 16113084 | 103.43 | 6.96E-04 | rs2271545 | 1.76E-04 |
| DBP, diastolic blood pressure; CHR, chromosome. | | | | | | | | |
