# Supplementary material for: Heritability and genome‐wide association study of blood pressure in Chinese adult twins
Source: Mol Genet Genomic Med. 2021 Sep 29;9(11):e1828. doi: 10.1002/mgg3.1828 (PMC8606211; doi:10.1002/mgg3.1828)
Supplement: Supplementary file 7 — Table S7 [file MGG3-9-e1828-s002.doc]

| Supplemental Table 7 The top 20 genes from VEGAS2 gene-based analysis showing the strongest association with PP level in typed GWAS data | | | | | | | | |
| --- | --- | --- | --- | --- | --- | --- | --- | --- |
| CHR | Gene | Numbers of SNPs | Start position | Stop position | Gene-based test statistic | Gene *P*-value | Top-SNP | Top-SNP *P*-value |
| 19 | ZNF576 | 5 | 44100543 | 44104587 | 30.98 | 7.00E-06 | rs2240932 | 2.98E-04 |
| 14 | IPO4 | 9 | 24649424 | 24658124 | 65.74 | 6.10E-05 | rs11550452 | 2.02E-04 |
| 11 | OR8D2 | 4 | 124189157 | 124190093 | 33.99 | 9.20E-05 | rs74550564 | 5.01E-05 |
| 14 | ZFHX2 | 16 | 23990063 | 24020858 | 113.23 | 9.50E-05 | rs7144469 | 9.06E-05 |
| 14 | THTPA | 27 | 23980968 | 24028790 | 155.32 | 1.00E-04 | rs7144469 | 9.06E-05 |
| 3 | THRB | 317 | 24158644 | 24536313 | 719.45 | 3.13E-04 | rs6777133 | 6.64E-05 |
| 1 | SNRNP40 | 10 | 31732414 | 31769644 | 60.23 | 3.40E-04 | rs2292164 | 4.29E-04 |
| 9 | KIF12 | 2 | 116853917 | 116861337 | 15.29 | 4.87E-04 | rs77229224 | 4.85E-04 |
| 19 | RINL | 9 | 39358471 | 39368919 | 51.13 | 5.22E-04 | rs10424892 | 3.35E-04 |
| 14 | ATG14 | 9 | 55833108 | 55878576 | 51.72 | 5.45E-04 | rs199596610 | 3.12E-04 |
| 1 | TACSTD2 | 5 | 59041094 | 59043166 | 34.14 | 6.49E-04 | rs7333 | 2.26E-03 |
| 23 | ELF4 | 5 | 129198894 | 129244688 | 35.59 | 7.01E-04 | rs209990 | 1.38E-04 |
| 1 | ITLN2 | 6 | 160914815 | 160924589 | 35.71 | 7.08E-04 | rs955615 | 4.50E-04 |
| 12 | ORMDL2 | 2 | 56211805 | 56214959 | 14.73 | 7.14E-04 | rs56108400 | 2.66E-03 |
| 2 | ABHD1 | 3 | 27346656 | 27353680 | 29.98 | 7.60E-04 | rs4665947 | 4.56E-04 |
| 23 | BMP15 | 4 | 50653734 | 50659641 | 27.40 | 7.68E-04 | rs3897937 | 1.42E-03 |
| 9 | LCN1 | 4 | 138413283 | 138418386 | 25.38 | 7.81E-04 | rs34145173 | 1.06E-03 |
| 20 | LOC729296 | 14 | 58662740 | 58676442 | 84.61 | 7.85E-04 | rs13433360 | 5.05E-04 |
| 5 | LOC101928093 | 6 | 172182505 | 172189565 | 39.76 | 8.27E-04 | rs4867688 | 3.31E-04 |
| 7 | ZDHHC4 | 5 | 6617064 | 6628610 | 28.62 | 9.36E-04 | rs11559146 | 6.48E-04 |
| PP, pulse pressure; CHR, chromosome. | | | | | | | | |
