# Supplementary material for: Heritability and genome‐wide association study of blood pressure in Chinese adult twins
Source: Mol Genet Genomic Med. 2021 Sep 29;9(11):e1828. doi: 10.1002/mgg3.1828 (PMC8606211; doi:10.1002/mgg3.1828)
Supplement: Supplementary file 8 — Table S8 [file MGG3-9-e1828-s009.doc]

| Supplemental Table 8 The top 20 genes from VEGAS2 gene-based analysis showing the strongest association with MAP level in typed GWAS data | | | | | | | | |
| --- | --- | --- | --- | --- | --- | --- | --- | --- |
| CHR | Gene | Numbers of SNPs | Start position | Stop position | Gene-based test statistic | Gene *P*-value | Top-SNP | Top-SNP *P*-value |
| 13 | LINC00346 | 6 | 111516333 | 111522655 | 57.75 | 2.90E-05 | rs9588287 | 9.54E-05 |
| 11 | SLC37A4 | 7 | 118895060 | 118901616 | 50.39 | 7.40E-05 | rs11006 | 4.52E-05 |
| 11 | TRAPPC4 | 3 | 118889240 | 118894385 | 38.15 | 1.19E-04 | rs3802884 | 2.41E-05 |
| 6 | SLC35B2 | 5 | 44221837 | 44225627 | 28.75 | 1.40E-04 | rs3734707 | 3.54E-04 |
| 13 | BORA | 14 | 73301886 | 73330336 | 87.46 | 1.60E-04 | rs9543104 | 1.41E-04 |
| 21 | TFF2 | 3 | 43766466 | 43771208 | 31.77 | 1.88E-04 | rs751143 | 4.97E-04 |
| 2 | FEV | 2 | 219845808 | 219850379 | 27.41 | 2.05E-04 | rs2301296 | 1.84E-04 |
| 19 | ZNF682 | 21 | 20115226 | 20150277 | 123.76 | 2.34E-04 | rs11085292 | 7.88E-05 |
| 2 | GTF2A1L | 32 | 48844918 | 48906751 | 285.12 | 2.45E-04 | rs34003293 | 1.51E-05 |
| 2 | LINC01158 | 14 | 105421882 | 105467934 | 89.25 | 2.75E-04 | rs4851716 | 6.84E-05 |
| 1 | ANGPTL7 | 9 | 11249345 | 11256038 | 41.07 | 3.21E-04 | rs28918368 | 4.69E-04 |
| 1 | FMO9P | 8 | 166573152 | 166594473 | 68.00 | 3.38E-04 | rs12039131 | 1.79E-04 |
| 8 | ASAP1 | 117 | 131064350 | 131455906 | 617.06 | 3.45E-04 | rs62524640 | 6.37E-05 |
| 7 | EGFR | 110 | 55086724 | 55275031 | 398.09 | 3.62E-04 | rs845560 | 7.70E-04 |
| 2 | LINC01114 | 10 | 105363094 | 105374177 | 60.25 | 3.95E-04 | rs61013045 | 1.14E-04 |
| 11 | KCNK7 | 5 | 65360325 | 65363467 | 30.08 | 4.22E-04 | rs76353556 | 2.24E-03 |
| 15 | LINC00926 | 9 | 57592562 | 57599967 | 70.84 | 4.50E-04 | rs10152152 | 3.49E-04 |
| 13 | MZT1 | 11 | 73282494 | 73301938 | 71.63 | 4.52E-04 | rs7327295 | 6.69E-04 |
| 15 | BCL2A1 | 8 | 80253231 | 80263643 | 55.54 | 4.62E-04 | rs6495460 | 7.50E-04 |
| 1 | AP4B1 | 7 | 114437370 | 114447741 | 34.60 | 4.64E-04 | rs1217397 | 8.49E-04 |
| MAP, mean arterial pressure; CHR, chromosome. | | | | | | | | |
