# Supplementary material for: Heritability and genome‐wide association study of blood pressure in Chinese adult twins
Source: Mol Genet Genomic Med. 2021 Sep 29;9(11):e1828. doi: 10.1002/mgg3.1828 (PMC8606211; doi:10.1002/mgg3.1828)
Supplement: Supplementary file 9 — Table S9 [file MGG3-9-e1828-s006.doc]

| **Supplemental Table 9** Common genes among SBP, DBP, PP and MAP | | | | | | |
| --- | --- | --- | --- | --- | --- | --- |
| Gene | CHR | Numbers of SNPs | *P* value in SBP | *P* value in DBP | *P* value in PP | *P* value in MAP |
| THRB | 3 | 317 | 4.50E-05 | 1.81E-02 | 3.13E-04 | 7.57E-04 |
| PSMB3 | 17 | 5 | 1.03E-04 | 2.06E-02 | 5.13E-03 | 5.11E-04 |
| OR8D1 | 11 | 4 | 2.23E-04 | 1.33E-02 | 5.91E-03 | 5.95E-04 |
| SLC35B2 | 6 | 5 | 2.24E-04 | 3.77E-03 | 5.61E-03 | 1.40E-04 |
| BCL2A1 | 15 | 8 | 2.42E-04 | 4.01E-03 | 2.44E-03 | 4.62E-04 |
| ANGPTL7 | 1 | 9 | 3.17E-04 | 1.36E-03 | 1.67E-02 | 3.21E-04 |
| ZNF682 | 19 | 21 | 4.36E-04 | 9.70E-04 | 4.66E-02 | 2.34E-04 |
| ZNF580 | 19 | 3 | 4.74E-04 | 2.73E-03 | 3.41E-02 | 5.50E-04 |
| GTF2A1L | 2 | 32 | 4.90E-04 | 1.15E-03 | 1.46E-02 | 2.45E-04 |
| MBD3 | 19 | 4 | 4.90E-04 | 1.88E-02 | 1.03E-02 | 2.11E-03 |
| GHRHR | 7 | 21 | 5.19E-04 | 2.53E-02 | 1.98E-03 | 1.92E-03 |
| TMEM220 | 17 | 18 | 5.45E-04 | 2.80E-02 | 1.30E-02 | 2.38E-03 |
| PFKFB3 | 10 | 131 | 5.77E-04 | 2.63E-02 | 1.07E-02 | 2.00E-03 |
| C10orf53 | 10 | 30 | 7.39E-04 | 1.47E-02 | 1.37E-02 | 1.16E-03 |
| NMS | 2 | 6 | 7.54E-04 | 1.48E-02 | 1.74E-02 | 1.69E-03 |
| BORA | 13 | 14 | 7.93E-04 | 1.02E-03 | 3.73E-02 | 1.60E-04 |
| FAM71E2 | 19 | 10 | 8.83E-04 | 4.18E-03 | 3.51E-02 | 6.85E-04 |
| MAGI2-AS2 | 7 | 2 | 1.23E-03 | 1.27E-02 | 4.89E-02 | 9.70E-04 |
| OR1L3 | 9 | 6 | 1.66E-03 | 2.24E-03 | 3.78E-02 | 1.28E-03 |
| NAP1L1 | 12 | 11 | 1.80E-03 | 2.75E-02 | 9.39E-03 | 3.18E-03 |
| WIF1 | 12 | 17 | 1.83E-03 | 1.91E-02 | 3.40E-02 | 3.60E-03 |
| ADPRM | 17 | 5 | 1.94E-03 | 4.84E-02 | 1.93E-02 | 5.30E-03 |
| C16orf97 | 16 | 23 | 2.02E-03 | 4.64E-02 | 1.12E-02 | 4.56E-03 |
| HAPLN1 | 5 | 51 | 2.43E-03 | 3.46E-02 | 2.96E-03 | 9.80E-03 |
| HS6ST1 | 2 | 26 | 2.69E-03 | 1.52E-02 | 1.81E-02 | 3.54E-03 |
| RUFY3 | 4 | 24 | 2.77E-03 | 3.85E-02 | 3.17E-02 | 4.12E-03 |
| TRIO | 5 | 106 | 2.83E-03 | 2.96E-02 | 1.93E-02 | 7.33E-03 |
| FLVCR2 | 14 | 34 | 3.32E-03 | 1.01E-02 | 4.35E-02 | 2.82E-03 |
| SLC22A1 | 6 | 40 | 3.41E-03 | 5.60E-03 | 2.01E-02 | 3.54E-03 |
| ATP6V0E2 | 7 | 7 | 3.55E-03 | 1.43E-02 | 2.19E-02 | 4.59E-03 |
| SFXN4 | 10 | 17 | 3.68E-03 | 2.18E-02 | 9.20E-03 | 5.76E-03 |
| CHST14 | 15 | 3 | 3.75E-03 | 7.86E-04 | 8.90E-03 | 1.48E-03 |
| MRPL18 | 6 | 5 | 4.49E-03 | 3.14E-02 | 3.99E-02 | 4.24E-03 |
| KLF17 | 1 | 15 | 4.75E-03 | 3.94E-02 | 2.52E-02 | 7.95E-03 |
| DNAJC17 | 15 | 12 | 5.45E-03 | 1.89E-02 | 2.89E-02 | 7.08E-03 |
| TMEM173 | 5 | 7 | 5.57E-03 | 3.91E-02 | 2.79E-02 | 7.13E-03 |
| AS3MT | 10 | 12 | 6.10E-03 | 1.98E-02 | 3.20E-02 | 8.14E-03 |
| QTRTD1 | 3 | 11 | 7.57E-03 | 3.89E-02 | 4.40E-02 | 1.42E-02 |
| CACNA1C-AS1 | 12 | 7 | 8.24E-03 | 3.79E-02 | 2.33E-02 | 1.63E-02 |
| GPR132 | 14 | 8 | 1.00E-02 | 3.60E-02 | 4.65E-02 | 1.03E-02 |
| ARG1 | 6 | 5 | 1.04E-02 | 1.36E-02 | 4.80E-02 | 6.82E-03 |
| DHRS4-AS1 | 14 | 2 | 1.26E-02 | 3.96E-02 | 4.79E-02 | 1.44E-02 |
| GPR1 | 2 | 21 | 1.73E-02 | 3.69E-02 | 9.79E-03 | 3.75E-02 |
| EPHA4 | 2 | 95 | 1.75E-02 | 4.85E-02 | 4.12E-02 | 1.96E-02 |
| PHGR1 | 15 | 6 | 2.23E-02 | 7.67E-03 | 4.01E-02 | 6.71E-03 |
| CNGB1 | 16 | 78 | 2.68E-02 | 2.24E-02 | 2.73E-02 | 2.11E-02 |
| MIR6165_5 | 5 | 3 | 3.70E-02 | 1.28E-02 | 4.75E-02 | 3.12E-02 |
| ATP6V0B | 1 | 3 | 4.14E-02 | 4.01E-02 | 4.78E-02 | 3.01E-02 |
| STON1-GTF2A1L | 2 | 167 | 9.60E-04 | 5.67E-03 | 3.03E-02 | 7.07E-04 |
| LOC102724596 | 17 | 8 | 2.35E-04 | 1.02E-02 | 7.31E-03 | 7.64E-04 |
| LINC00939 | 12 | 26 | 1.42E-03 | 9.58E-03 | 2.42E-02 | 1.23E-03 |
| LOC102467079 | 16 | 6 | 6.66E-04 | 3.51E-02 | 2.89E-03 | 2.24E-03 |
| LOC102724153 | 14 | 7 | 6.84E-04 | 2.57E-02 | 3.69E-03 | 2.54E-03 |
| SBP: systolic blood pressure; DBP: diastolic blood pressure; PP: pulse pressure; MAP: mean arterial pressure; CHR: chromosome. | | | | | | |
