# Supplementary material for: Heritability and genome‐wide association study of blood pressure in Chinese adult twins
Source: Mol Genet Genomic Med. 2021 Sep 29;9(11):e1828. doi: 10.1002/mgg3.1828 (PMC8606211; doi:10.1002/mgg3.1828)
Supplement: Supplementary file 10 — Table S10 [file MGG3-9-e1828-s001.doc]

| **Supplemental Table 10** The top 20 pathway results-KEGG, Reactome, and Biocarta (emp-P < 0.05) using PASCAL program for SBP level in GWAS data | | | | |
| --- | --- | --- | --- | --- |
| Pathway | chisq-P | emp-P | -log(chisqP) | –log(empP) |
| REACTOME_SIGNALING_BY_ERBB4 | 1.67E-04 | 1.52E-04 | 3.78 | 3.82 |
| REACTOME_CELL_JUNCTION_ORGANIZATION | 1.07E-03 | 3.93E-04 | 2.97 | 3.41 |
| REACTOME_CELL_CELL_COMMUNICATION | 1.26E-03 | 5.20E-04 | 2.90 | 3.28 |
| REACTOME_METABOLISM_OF_MRNA | 2.97E-03 | 5.40E-04 | 2.53 | 3.27 |
| BIOCARTA_EGFR_SMRTE_PATHWAY | 7.88E-04 | 6.30E-04 | 3.10 | 3.20 |
| KEGG_PRION_DISEASES | 1.21E-03 | 7.00E-04 | 2.92 | 3.15 |
| REACTOME_ADAPTIVE_IMMUNE_SYSTEM | 1.84E-03 | 7.80E-04 | 2.73 | 3.11 |
| BIOCARTA_INFLAM_PATHWAY | 2.84E-03 | 8.80E-04 | 2.55 | 3.06 |
| BIOCARTA_ERYTH_PATHWAY | 2.84E-03 | 9.20E-04 | 2.55 | 3.04 |
| KEGG_TYROSINE_METABOLISM | 3.61E-03 | 1.29E-03 | 2.44 | 2.89 |
| REACTOME_PKA_MEDIATED_PHOSPHORYLATION_OF_CREB | 1.26E-03 | 1.29E-03 | 2.90 | 2.89 |
| KEGG_BASAL_TRANSCRIPTION_FACTORS | 1.38E-03 | 1.33E-03 | 2.86 | 2.88 |
| REACTOME_RAP1_SIGNALLING | 1.26E-03 | 1.35E-03 | 2.90 | 2.87 |
| REACTOME_NUCLEAR_RECEPTOR_TRANSCRIPTION_PATHWAY | 7.43E-03 | 1.64E-03 | 2.13 | 2.79 |
| KEGG_DILATED_CARDIOMYOPATHY | 1.96E-03 | 1.72E-03 | 2.71 | 2.76 |
| BIOCARTA_SKP2E2F_PATHWAY | 1.85E-03 | 1.73E-03 | 2.73 | 2.76 |
| REACTOME_PROLACTIN_RECEPTOR_SIGNALING | 1.85E-03 | 1.76E-03 | 2.73 | 2.75 |
| REACTOME_NUCLEAR_SIGNALING_BY_ERBB4 | 1.85E-03 | 1.85E-03 | 2.73 | 2.73 |
| REACTOME_REGULATION_OF_INSULIN_SECRETION | 2.23E-03 | 1.97E-03 | 2.65 | 2.71 |
| REACTOME_HORMONE_LIGAND_BINDING_RECEPTORS | 2.07E-03 | 2.04E-03 | 2.68 | 2.69 |
| chisq-*P*, Chi-square *p*-value. Chi-squared method (gene-score *p*-value were ranked and transformed to a uniform distribution, these values were then transformed by a chi-square quantile function, and summed).  emp-*P*, empirical *p*-value. Empirical sampling method (gene-scores are transformed with chi-square quantile function and summed, then Monte Carlo estimate of the *p*-values were obtained by sampling random sets of the same size). | | | | |
