# Supplementary material for: Heritability and genome‐wide association study of blood pressure in Chinese adult twins
Source: Mol Genet Genomic Med. 2021 Sep 29;9(11):e1828. doi: 10.1002/mgg3.1828 (PMC8606211; doi:10.1002/mgg3.1828)
Supplement: Supplementary file 11 — Table S11 [file MGG3-9-e1828-s008.doc]

| **Supplemental Table 11** The top 20 pathway results-KEGG, Reactome, and Biocarta (emp-P < 0.05) using PASCAL program for DBP level in GWAS data | | | | |
| --- | --- | --- | --- | --- |
| Pathway | chisq-P | emp-P | -log(chisqP) | –log(empP) |
| BIOCARTA_ERYTH_PATHWAY | 1.32E-03 | 5.20E-04 | 2.88 | 3.28 |
| BIOCARTA_INFLAM_PATHWAY | 1.32E-03 | 6.90E-04 | 2.88 | 3.16 |
| BIOCARTA_EGFR_SMRTE_PATHWAY | 9.23E-04 | 7.50E-04 | 3.03 | 3.12 |
| REACTOME_GAB1_SIGNALOSOME | 9.23E-04 | 7.80E-04 | 3.03 | 3.11 |
| BIOCARTA_CARDIACEGF_PATHWAY | 9.23E-04 | 8.30E-04 | 3.03 | 3.08 |
| REACTOME_GRB2_EVENTS_IN_ERBB2_SIGNALING | 9.23E-04 | 8.50E-04 | 3.03 | 3.07 |
| REACTOME_EGFR_DOWNREGULATION | 9.23E-04 | 8.60E-04 | 3.03 | 3.07 |
| REACTOME_SHC1_EVENTS_IN_EGFR_SIGNALING | 9.23E-04 | 8.60E-04 | 3.03 | 3.07 |
| REACTOME_PI3K_EVENTS_IN_ERBB2_SIGNALING | 9.23E-04 | 9.00E-04 | 3.03 | 3.05 |
| REACTOME_SIGNALING_BY_CONSTITUTIVELY_ACTIVE_EGFR | 9.23E-04 | 9.10E-04 | 3.03 | 3.04 |
| BIOCARTA_SPRY_PATHWAY | 9.23E-04 | 9.20E-04 | 3.03 | 3.04 |
| BIOCARTA_TEL_PATHWAY | 9.23E-04 | 9.60E-04 | 3.03 | 3.02 |
| KEGG_DORSO_VENTRAL_AXIS_FORMATION | 9.23E-04 | 9.80E-04 | 3.03 | 3.01 |
| REACTOME_IL_RECEPTOR_SHC_SIGNALING | 1.13E-03 | 1.01E-03 | 2.95 | 3.00 |
| BIOCARTA_EGF_PATHWAY | 9.23E-04 | 1.04E-03 | 3.03 | 2.98 |
| REACTOME_NCAM1_INTERACTIONS | 1.10E-03 | 1.05E-03 | 2.96 | 2.98 |
| BIOCARTA_TH1TH2_PATHWAY | 1.13E-03 | 1.18E-03 | 2.95 | 2.93 |
| REACTOME_IL_3_5_AND_GM_CSF_SIGNALING | 1.59E-03 | 1.25E-03 | 2.80 | 2.90 |
| BIOCARTA_TOB1_PATHWAY | 1.13E-03 | 1.31E-03 | 2.95 | 2.88 |
| BIOCARTA_CBL_PATHWAY | 2.46E-03 | 1.50E-03 | 2.61 | 2.82 |
| chisq-*P*, Chi-square *p*-value. Chi-squared method (gene-score *p*-value were ranked and transformed to a uniform distribution, these values were then transformed by a chi-square quantile function, and summed).  emp-*P*, empirical *p*-value. Empirical sampling method (gene-scores are transformed with chi-square quantile function and summed, then Monte Carlo estimate of the *p*-values were obtained by sampling random sets of the same size). | | | | |
