# Supplementary material for: Heritability and genome‐wide association study of blood pressure in Chinese adult twins
Source: Mol Genet Genomic Med. 2021 Sep 29;9(11):e1828. doi: 10.1002/mgg3.1828 (PMC8606211; doi:10.1002/mgg3.1828)
Supplement: Supplementary file 12 — Table S12 [file MGG3-9-e1828-s013.doc]

| **Supplemental Table 12** The top 20 pathway results-KEGG, Reactome, and Biocarta (emp-P < 0.05) using PASCAL program for PP level in GWAS data | | | | |
| --- | --- | --- | --- | --- |
| Pathway | chisq-P | emp-P | -log(chisqP) | –log(empP) |
| KEGG_TYROSINE_METABOLISM | 4.89E-04 | 8.10E-05 | 3.10 | 3.17 |
| BIOCARTA_MTA3_PATHWAY | 6.30E-04 | 5.70E-04 | 2.75 | 3.15 |
| REACTOME_DOWNSTREAM_SIGNALING_EVENTS_OF_B_CELL_RECEPTOR_BCR | 2.94E-03 | 6.60E-04 | 2.53 | 3.12 |
| BIOCARTA_EGFR_SMRTE_PATHWAY | 7.88E-04 | 6.70E-04 | 2.79 | 3.11 |
| KEGG_COLORECTAL_CANCER | 1.78E-03 | 7.10E-04 | 2.37 | 3.03 |
| REACTOME_SIGNALING_BY_THE_B_CELL_RECEPTOR_BCR | 2.94E-03 | 7.50E-04 | 2.53 | 3.03 |
| REACTOME_TRANSLATION | 1.64E-03 | 7.80E-04 | 2.44 | 3.00 |
| REACTOME_ANTIGEN_PROCESSING_UBIQUITINATION_PROTEASOME_DEGRADATION | 4.27E-03 | 9.30E-04 | 2.51 | 3.00 |
| REACTOME_IMMUNE_SYSTEM | 2.96E-03 | 9.30E-04 | 2.88 | 2.91 |
| REACTOME_ADAPTIVE_IMMUNE_SYSTEM | 3.63E-03 | 9.90E-04 | 2.37 | 2.91 |
| KEGG_SPHINGOLIPID_METABOLISM | 3.11E-03 | 1.01E-03 | 2.88 | 2.89 |
| REACTOME_RAP1_SIGNALLING | 1.31E-03 | 1.23E-03 | 2.67 | 2.89 |
| REACTOME_CLASS_I_MHC_MEDIATED_ANTIGEN_PROCESSING_PRESENTATION | 4.27E-03 | 1.24E-03 | 2.39 | 2.84 |
| REACTOME_EFFECTS_OF_PIP2_HYDROLYSIS | 1.31E-03 | 1.28E-03 | 2.73 | 2.76 |
| REACTOME_ANTIGEN_PROCESSING_CROSS_PRESENTATION | 2.13E-03 | 1.30E-03 | 2.32 | 2.74 |
| REACTOME_NUCLEAR_SIGNALING_BY_ERBB4 | 1.85E-03 | 1.74E-03 | 2.73 | 2.72 |
| REACTOME_PLATELET_AGGREGATION_PLUG_FORMATION | 4.74E-03 | 1.82E-03 | 3.10 | 3.17 |
| BIOCARTA_P27_PATHWAY | 1.85E-03 | 1.84E-03 | 2.75 | 3.15 |
| REACTOME_PROLACTIN_RECEPTOR_SIGNALING | 1.85E-03 | 1.89E-03 | 2.53 | 3.12 |
| REACTOME_INTEGRIN_ALPHAIIB_BETA3_SIGNALING | 4.74E-03 | 1.93E-03 | 2.32 | 2.71 |
| chisq-*P*, Chi-square *p*-value. Chi-squared method (gene-score *p*-value were ranked and transformed to a uniform distribution, these values were then transformed by a chi-square quantile function, and summed).  emp-*P*, empirical *p*-value. Empirical sampling method (gene-scores are transformed with chi-square quantile function and summed, then Monte Carlo estimate of the *p*-values were obtained by sampling random sets of the same size). | | | | |
