# Supplementary material for: Heritability and genome‐wide association study of blood pressure in Chinese adult twins
Source: Mol Genet Genomic Med. 2021 Sep 29;9(11):e1828. doi: 10.1002/mgg3.1828 (PMC8606211; doi:10.1002/mgg3.1828)
Supplement: Supplementary file 13 — Table S13 [file MGG3-9-e1828-s004.doc]

| **Supplemental Table 13** The top 20 pathway results-KEGG, Reactome, and Biocarta (emp-P < 0.05) using PASCAL program for MAP level in GWAS data | | | | |
| --- | --- | --- | --- | --- |
| Pathway | chisq-P | emp-P | -log(chisqP) | –log(empP) |
| BIOCARTA_ERYTH_PATHWAY | 3.72E-04 | 8.80E-05 | 3.43 | 4.06 |
| BIOCARTA_INFLAM_PATHWAY | 3.72E-04 | 9.70E-05 | 3.43 | 4.01 |
| REACTOME_SIGNALING_BY_ERBB4 | 8.90E-04 | 1.61E-04 | 3.05 | 3.79 |
| BIOCARTA_AT1R_PATHWAY | 1.92E-03 | 4.32E-04 | 2.72 | 3.36 |
| BIOCARTA_AGR_PATHWAY | 1.92E-03 | 4.45E-04 | 2.72 | 3.35 |
| REACTOME_SIGNAL_TRANSDUCTION_BY_L1 | 1.92E-03 | 6.00E-04 | 2.72 | 3.22 |
| REACTOME_CASPASE_MEDIATED_CLEAVAGE_OF_CYTOSKELETAL_PROTEINS | 7.08E-04 | 7.60E-04 | 3.15 | 3.12 |
| KEGG_EPITHELIAL_CELL_SIGNALING_IN_HELICOBACTER_PYLORI_INFECTION | 2.51E-03 | 8.20E-04 | 2.60 | 3.09 |
| BIOCARTA_EGFR_SMRTE_PATHWAY | 7.88E-04 | 8.40E-04 | 3.10 | 3.08 |
| REACTOME_NUCLEAR_RECEPTOR_TRANSCRIPTION_PATHWAY | 2.87E-03 | 1.07E-03 | 2.54 | 2.97 |
| KEGG_GNRH_SIGNALING_PATHWAY | 3.96E-03 | 1.25E-03 | 2.40 | 2.90 |
| REACTOME_HOST_INTERACTIONS_OF_HIV_FACTORS | 2.22E-03 | 1.27E-03 | 2.65 | 2.90 |
| REACTOME_EGFR_DOWNREGULATION | 1.85E-03 | 1.55E-03 | 2.73 | 2.81 |
| KEGG_DORSO_VENTRAL_AXIS_FORMATION | 1.85E-03 | 1.63E-03 | 2.73 | 2.78 |
| BIOCARTA_CBL_PATHWAY | 4.72E-03 | 1.66E-03 | 2.33 | 2.78 |
| REACTOME_L1CAM_INTERACTIONS | 5.70E-03 | 1.66E-03 | 2.24 | 2.78 |
| BIOCARTA_CARDIACEGF_PATHWAY | 1.85E-03 | 1.74E-03 | 2.73 | 2.76 |
| REACTOME_PI3K_EVENTS_IN_ERBB2_SIGNALING | 1.85E-03 | 1.74E-03 | 2.73 | 2.76 |
| BIOCARTA_SPRY_PATHWAY | 1.85E-03 | 1.77E-03 | 2.73 | 2.75 |
| REACTOME_SHC1_EVENTS_IN_EGFR_SIGNALING | 1.85E-03 | 1.81E-03 | 2.73 | 2.74 |
| chisq-*P*, Chi-square *p*-value. Chi-squared method (gene-score *p*-value were ranked and transformed to a uniform distribution, these values were then transformed by a chi-square quantile function, and summed).  emp-*P*, empirical *p*-value. Empirical sampling method (gene-scores are transformed with chi-square quantile function and summed, then Monte Carlo estimate of the *p*-values were obtained by sampling random sets of the same size). obtained by sampling random sets of the same size). | | | | |
